# Updated Insights Into the Mechanism of Salt‐Induced Aggregation‐Based Single‐Molecule Surface‐Enhanced Raman Spectroscopy

**DOI:** 10.1002/advs.202417025

**Published:** 2025-03-13

**Authors:** Lingwei Li, Ruiyuan Zhang, Yu Guo, Jiacheng Ge, Si Lan, Feng Tian, Yi‐Tao Long, Hongjun You, Jixiang Fang

**Affiliations:** ^1^ Key Laboratory of Biomedical Information Engineering of Ministry of Education School of Life Science and Technology Xi'an Jiaotong University Xi'an Shaanxi 710049 P. R. China; ^2^ Herbert Gleiter Institute of Nanoscience School of Materials Science and Engineering Nanjing University of Science & Technology Nanjing Jiangsu 210094 P. R. China; ^3^ Shanghai Synchrotron Radiation Facility Shanghai Advanced Research Institute Chinese Academy of Sciences Shanghai 201204 P. R. China; ^4^ School of Chemistry and Chemical Engineering State Key Laboratory of Analytical Chemistry for Life Science Nanjing University Nanjing Jiangsu 210023 P. R. China

**Keywords:** Ag colloid, aggregation agent, single molecule, surface‐enhanced Raman spectroscopy

## Abstract

Since its discovery in 1997, the single molecule surface‐enhanced Raman spectroscopy (SM‐SERS) has attracted wide interest owing to its enormous potential in many fields. However, the commercialized applications of SM‐SERS are still limited by the lack of a clear understanding of the relevant mechanism in the famous SM‐SERS experiments. In this study, a salt‐gradient model is proposed to deeply investigate the physical nature and update insights into the morphological, structural, and component evolution processes of Ag NPs from dispersed nanostructures to aggregation states in the salt‐induced aggregation SERS strategy. A gradient interface is observed, where an ultrahigh sensitivity approaching a single molecule level, has been achieved in Ag colloidal system. An unusual dissolution of Ag, the release of Ag^+^ ions from Ag NPs, and the final precipitation of AgCl can be evidenced. Thus, except for aggregation effect, the active AgCl packaging shell on the surface of Ag NPs remarkably improves the SERS property. This work not only reveals the physics processes and nature of SM‐SERS but also offers a new way to exploit the SM‐SERS into practical applications by means of designing different surface states of NPs and various activation compositions to meet diverse molecule systems.

## Graphical abstract

A salt‐gradient model is proposed to investigate the physical nature and update insights into the single molecule surface‐enhanced Raman spectroscopy (SM‐SERS). The release of Ag^+^ ions from the Ag NPs and the final formation of precipitate of AgCl with Cl^‐^ on the surface of the aggregated Ag NPs plays as the active and packaging shell to improve the SM‐SERS property.

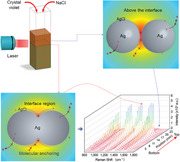

## 1 Introduction

Since the first discovery of the surface‐enhanced Raman scattering (SERS) effect in 1974,^[^ ^1^ ^]^ particularly the great breakthroughs in the single‐molecule SERS (SM‐SERS) in 1997,^[^ ^2^, ^3^ ^]^ the interest of the scientific community in SERS has been rekindled immediately owing to this powerful combination of single‐molecule sensitivity and spectroscopic fingerprinting in many fields.^[^ ^4^, ^5^, ^6^, ^7^ ^]^ In the last three decades, as the in‐depth understanding of enhancement mechanism in SERS, the basic scientific question regarding the interaction between light and nanostructures has been investigated intensively.^[^ ^8^, ^9^, ^10^, ^11^ ^]^ The electromagnetic enhancement and hot spot effect have been considered as the main enhancement mechanism in SERS.^[^ ^12^, ^13^, ^14^ ^]^ Meanwhile, along with the prosperity of nanotechnology, an immense amount of plasmonic nanostructures used as SERS substrates have been exploited.^[^ ^15^, ^16^, ^17^, ^18^, ^19^ ^]^ However, up to now, all seems to imply that it is still very difficult to reach a stable detection using diverse nano‐structural SERS substrates to an ultra‐low limit of detection (LOD), e.g., ≈10^−14^ m, relying only on the electromagnetic hot spot effect.

In fact, the famous SM‐SERS phenomenon reported in 1997 occurred in narrow and limited conditions (Figure S1, Supporting Information) in the colloidal nanoparticle (NP) system, e.g., the use of Ag NPs and low concentration of NaCl as SERS substrate and activation agent, respectively.^[^ ^2^, ^3^ ^]^ After the early reports of SM‐SERS, the following studies using atomic force microscopy (AFM), transmission electron microscopy (TEM), isotopologues, and super‐resolution optical imaging,^[^ ^20^, ^21^, ^22^, ^23^ ^]^ successfully verified the necessity of the aggregation of Ag NPs in achieving SM‐SERS property. Particularly, Doering and Nie et al. carried out an important investigation on the roles of surface active sites and chemical enhancement in SM‐SERS using an integrated flow injection and ultrasensitive optical imaging/spectroscopy system.^[^ ^24^ ^]^ In current commercial colloidal aggregation‐based SERS detection, a high concentration of halide salt is always used as an aggregation agent, which destroys the electric double layer of Au or Ag NPs and induces the aggregation of NPs.^[^ ^25^, ^26^ ^]^ However, people still do not know its mechanism where a relative lower concentration of halide salt may contribute to an enhanced SERS activity. In addition, the active effect of halide ions on the Ag NPs seems to have been ignored in the colloidal aggregation‐based SERS detection in previous studies.^[^ ^27^ ^]^ Therefore, in current commercial applications, the superiority of SM‐SERS with ultrahigh sensitivity has not been brought into full play yet.

Herein, a salt‐gradient model was proposed to investigate the influence of salt concentrations on the aggregation behavior and structural evolution of Ag NPs and Au NPs. A concentration gradient of NaCl can be created by means of the free diffusion of dropping a small amount of NaCl solution into the colloid (Figure S2, Supporting Information). An interface can be observed due to the discrepancy in the aggregation structure of colloidal NPs, and this novel phenomenon was subsequently exploited for simple and versatile solution‐based SERS detection. Unexpectedly, an extremely strong SERS signal approaching the single‐molecule detection level can be generated at the interface region of Ag colloidal system (the calculation of a single molecule shown in the Supporting Information). The influence of salt concentrations on the morphology, structure, composition, and their evolution of colloidal NPs hence on SERS property have been systemically studied and optimized. Unlike Au NPs system, a quite unusual phenomenon, i.e., an obvious structural evolution, from individual sphere to fusion, even irregularly connected agglomerates, can be observed in Ag NPs. A composition evolution process, i.e., the dissolution of Ag when adding NaCl and the release of Ag^+^ ions from Ag NPs, the precipitation of AgCl on the surface of the aggregation of Ag NPs, accompanying with the morphological and structural evolution, can be evidenced, which plays as the active shell to remarkably improve the SERS property. Therefore, this work not only reveals a clear picture regarding the physics processes and nature of SM‐SERS those have not been clarified in the salt‐aggregation SERS system, but also offers a way to exploit the SM‐SERS detection into the practical applications by means of designing different surface states of NPs.

## 2 Results and Discussion

In order to create a salt concentration gradient, a small amount of NaCl solution was dropped into the water without vibration using crystal violet (CV) as the color tracer. It is observed that the mixing solution of NaCl and CV falls to the bottom of the container and then diffuses upward, eventually reaching a steady state. Clearly, an obvious gradient distribution of CV molecules can be observed from the color changing (Figure S2A, Supporting Information) and its position‐dependent UV–vis spectra (Figure S2E, Supporting Information), where the characteristic absorption peaks of CV molecules increase with the position from top to bottom, revealing the concentration changing of CV. As a comparison, when only CV without NaCl were added to the water, by means of free diffusion, the CV finally dispersed uniformly in the whole container (Figure S2B, Supporting Information). Thus, a salt‐gradient distribution model can be formed by the current protocol.

**Figure 1:**
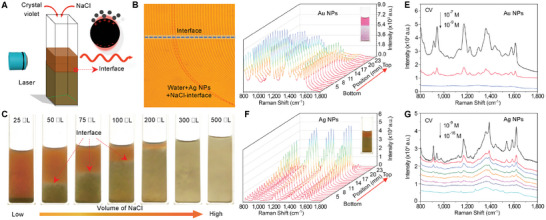
The salt‐gradient model system and the salt‐aggregation‐based SERS detection of CV. A) Schematic diagram of SERS detection at the interface of Ag colloid. B) Image of interference fringes obtained from Michelson interferometer measurement for Ag colloid with the interface by means of dropping NaCl into Ag NPs colloid, then without any vibration. C) The Ag NPs colloid with different adding volumes of NaCl solution. D) The position‐dependent SERS detection of CV (10^−8^ m) molecules in the Au NPs colloid with interface by adding 100 µL NaCl in the mixture of 500 µL Au NPs and 2 mL CV solution. E) The LOD of CV in the Au colloid system at the interface region. F) The position‐dependent SERS performance of CV molecules in the Ag NPs colloid with interface by adding 100 µL NaCl in the mixture of 500 µL Ag NPs and 2 mL CV solution. G) The LOD of CV in Ag colloid system detected at the interface region.

Next, a small amount of NaCl was dropped into the colloid solution of Ag NPs and Au NPs and an obvious interface was formed (Figure S2C,D, Supporting Information). Below the interface, the colloid changes its color to dark because of the aggregation of NPs. Above the interface, the color remains nearly unchanged. Michelson interferometer measurement has been used to further evaluate the distribution of NaCl in the colloid of Ag NPs, where its equal thickness interference fringe may indicate the change of the refraction indexes owing to the concentration diversity.^[^ ^28^ ^]^ After dropping NaCl solution into the colloid of Ag NPs, with shaking, the resulting interference fringes show a uniform character (Figure S3A, Supporting Information). However, without any vibration, the interference fringes display a bending feature below the interface region (**Figure** **1**B; Figure S3B, Supporting Information), revealing the gradient distribution of NaCl and various aggregation states of Ag NPs.

In the current commercial salt‐induced aggregation SERS strategy, an excessive aggregation agent and vibration of the mixing colloid are always employed. In this work, diverse NaCl amounts (from 25 to 500 µL) have been used. It is observed that the less feeding of NaCl into Ag and Au colloid, the lower position of the interface was formed. When the amount of NaCl exceeds a critical value, e.g., 200 µL, the interface can not be created both in Ag and Au colloid (Figure 1C; Figure S4, Supporting Information) even without vibration. Position‐dependent SERS signals of 10^−8^ m CV molecule for both Au and Ag colloidal with interface were detected. In Au NPs colloid, above the interface, the SERS signals can hardly be detected. The signals start to increase at the interface and then display consistent intensity at different positions below the interface (Figure 1D; Figure S5, Supporting Information). The LOD of CV in Au colloid is ≈10^−8^ m can be obtained for interface systems (Figure 1E). However, in Ag colloid, the SERS intensity of CV molecule progressively increases from the bottom, reaching its maximum at the interface region, and then swiftly declines above the interface (Figure 1F; Figure S6, Supporting Information). The best SERS signal was always detected at the interface region for the samples with different feeding amounts of NaCl (25–100 µL) (Figure S7, Supporting Information). Even if the integration time is reduced from 20 to 10 and 1 s, there is still the same significant signal variation with position in the interfaced Ag colloid (Figure S8, Supporting Information).

For the specimens of Ag colloidal without interface caused by excessive feeding of NaCl, e.g., 200 –500 µL, the SERS spectra display consistent signal intensity at different positions because of the distribution of excessive NaCl in the whole solution (Figure S9, Supporting Information). A higher detection sensitivity can always be achieved at the interface region compared to un‐interfaced solutions with excess NaCl in Ag colloid (Figure S10, Supporting Information). The LOD of 10^−12^ m and even 10^−15^ m for CV molecules was obtained with 50 and 100 µL of NaCl (Figure S10D, Supporting Information; Figure 1G). However, for the sample with 500 µL of NaCl, the LOD is only 10^−8^ m (Figure S10B, Supporting Information). An optimized detection capability with a LOD of fM for CV molecules can be obtained under the optimal volume of NaCl, i.e., 100 µL (Figure 1G; Figure S11, Supporting Information). The change in the statistical distribution of Raman signal for 100 SERS measurements from Gaussian to Poisson distribution when the concentration of CV decreased from 10^−14^ to 10^−15^ m indicates the detection of single molecule level was achieved (Figures S12 and S13, Supporting Information).^[^ ^2^ ^]^ By continuously collecting SERS signals of 10^−8^ m CV at the interface region of Ag colloids, the interfaced Ag colloid shows good stability in the SERS detection with stable signals that can be obtained within 25 min (Figure S14, Supporting Information). Repeatable SERS signals can be obtained in multiple detection batches of 10^−15^ m CV (Figure S15, Supporting Information), indicating excellent reproducibility of high sensitivity for this interfacial‐based SERS strategy.

**Figure 2:**
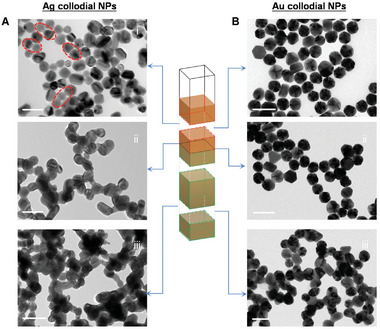
The position‐dependent TEM images of Ag NPs and Au NPs at gradient solution. A) The TEM images of Ag NPs at different positions of Ag colloid solution with the interface. B) The TEM images of Au NPs at different positions of Au colloid with the interface. 100 µL of NaCl was added to the colloid to form an interface. NPs at different positions of colloidal solution were distilled and dropped on the copper grids for TEM characterization. The scale bars are 100 nm.

In order to unequivocally investigate the origin of the discrepancy in SERS sensitivity at different positions, the representative position‐dependent TEM images of Ag NPs and Au NPs were obtained by carefully selecting the specimens at different regions. As is shown in **Figure** **2**A, an unexpected structural change with distinguishable morphology occurs on Ag NPs. At the top of the solution, the Ag NPs can keep the original spherical morphology like as‐prepared (Figure 2A‐i). As the position goes down, Ag NPs start to interconnect with each other slightly, instead of individual particles. At the interface region, the interconnection and fusion of Ag NPs become more pronounced (Figure 2A‐ii; Figure S16, Supporting Information). In the region at the bottom of the solution, as the concentration of NaCl increases, the interconnection and fusion of Ag NPs are more severe, forming irregularly connected agglomerates (Figure 2A‐iii). As a comparison, the morphology of Au NPs at different positions of solution were obtained. As shown in Figure 2B, Au NPs show a well‐dispersed state above the interface region (Figure 2B‐i). With the decrease of position, Au NPs gradually aggregate (Figure 2B‐ii). Obviously different with Ag NPs, no interconnection, and fusion can be observed even going down to the bottom region in the Au NPs system.

**Figure 3:**
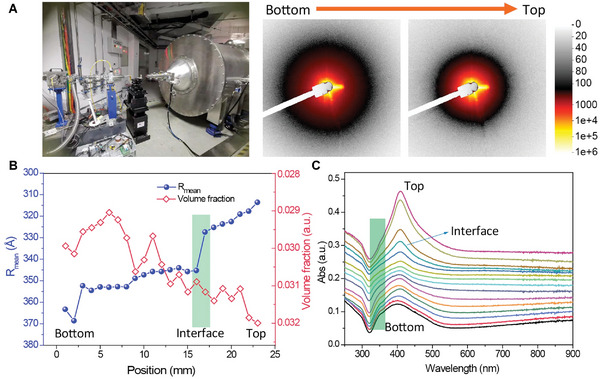
The characterization of Ag NPs at different positions of gradient solution. A) The SAXS characterize of Ag NPs colloid. B) The size distribution curves of Ag NPs at different positions. C) The position‐dependent UV–vis spectra of Ag NPs colloid.

To unambiguously investigate the aggregation degree of Ag NPs at different positions, in situ small‐angle X‐ray scattering (SAXS) was performed (**Figure** **3**A). The Q range in the SAXS detection is from 0.11697 to 1.5458 nm^−1^ (Figures S17 and S18). According to the size distribution fitting curves of Ag NPs as a function of different detection positions (Figure 3B; Figure S19, Supporting Information), one can find that, from the top to the bottom of the Ag colloid, average particle size gradually increases from 62 to 74 nm. A dramatic alteration in the size of Ag NPs is displayed at the interface region (Figure 3B), implying a sharp change in the aggregation state or structure of Ag NPs within this region. The volume fraction of Ag NPs gradually reduces from the top to the bottom of the gradient solution, which might be caused by the sedimentation of the aggregated Ag NPs.

The position‐dependent in situ UV–vis spectra of the Ag NPs colloid with the interface are shown in Figure 3C. The uppermost curve from the top region of the solution shows the characteristic absorption peak of Ag NPs at 409 nm. With the position downward, the intensity of the peaks gradually decreases, and the absorption peak of Ag NPs is gradually broadened with a slight blue shift from top to bottom. A peak at ≈350 nm appears and gradually becomes apparent from the interface to the bottom region. As a comparison, the UV spectra of Au NPs (Figure S20, Supporting Information) show a typical aggregation state with redshift character of the new peak initially from ≈800 to 1000 nm, and no peak is shown at low wavelength like the 350 nm peak in Ag NPs system. The above control experiments indicate that the simple aggregation effect is a dominant factor in the SERS performance of Au colloidal system. Above the interface, there is almost no SERS signal due to insufficient aggregation, and below the interface, no significant change is found in signal intensity as dependent on position.

The above results indicate that the huge enhanced SERS signal at the interface region of Ag colloid is quite an unusual phenomenon. According to previous literature and the finite‐difference time‐domain (FDTD) simulation (Figure S21, Supporting Information),^[^ ^10^, ^29^ ^]^ hot spots between adjacent NPs provide dramatic electromagnetic enhancement with the decrease of nanogap, as predicted in the classical model. When the over‐aggregated NPs gradually connect and even fuse together, the losses of nanogaps and hot spots drastically decrease in the electromagnetic field hence the SERS performance.^[^ ^30^ ^]^ However, in the current study, the Ag NPs were interconnected and fused together with an overlapping of ≈10 nm at the interface region (Figure 2A‐ii; Figure S16, Supporting Information), but an exceptionally superior SM‐SERS performance with a LOD of fM for CV molecule has been found (Figure S22, Supporting Information). This contradiction phenomenon reveals that, beyond the current electromagnetic mechanism, another crucial factor must be working to contribute to such superior SM‐SERS activity.

**Figure 4:**
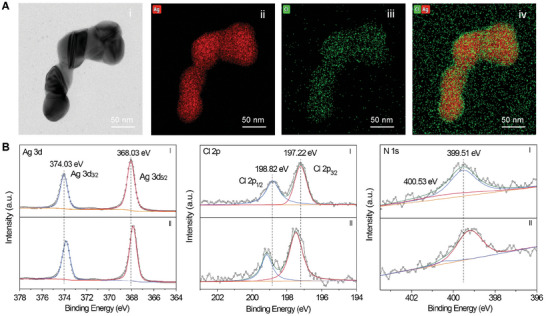
The EDX element mapping and the XPS spectra of aggregated Ag NPs. A) The typical TEM image and EDX element mapping of aggregated and fused Ag NPs. B) The XPS of Ag, Cl, and N elements of aggregated Ag NPs. NaCl was added to the mixture of Ag NPs colloid and CV solution. Ag NPs at the top (I), and bottom (II) region of the concentration gradient solution were taken out and dropped on the silicon slide after washing, then the XPS spectra were obtained after drying.

To further disclose the root cause of the structural evolution of Ag NPs at the different positions, the element mapping by means of energy dispersive X‐ray spectroscopy (EDX) was characterized. The Cl element with an increased amount on the surface of Ag NPs from 1.91% (at the top region) to 2.63% (at the bottom region) was found (Figure S23, Supporting Information). The typical morphology and composition of the connected Ag NPs have been further demonstrated with TEM image and EDX element mapping, as shown in **Figure** **4**A. The Ag (red) versus Cl (green) overlapping image (Figure 4A‐iv) displays the Cl element is highly localized on the surface of Ag NPs. Further, X‐ray photoelectron spectroscopy (XPS) was performed for Ag NPs at different positions. In Figure 4B, the peak of Ag 3d gradually shifts to lower binding energy from top to bottom of the gradient solution, which is probably attributed to the formation of Ag ion state, i.e., Ag^0^ to Ag^+^ ions. The peak of Cl 2p shifts to higher binding energy, relative to the perturbation by the electronic state of Ag.^[^ ^31^ ^]^ The gradual formation of Ag^+^ and increased interactions between Ag^+^ and Cl^−^ result in the shift of Ag 3d and Cl 2p. The peak of N 1s (from CV molecules) shifts to lower binding energy, further indicating the electronic interaction among Ag, Cl^−^, and the target molecule. Cl^−^ ions adsorb on the Ag surface by substituting citrate, then positive CV molecules are electrostatically attracted by the negative charge of Cl^−^ ions to form the Ag‐Cl‐CV complex.

**Figure 5:**
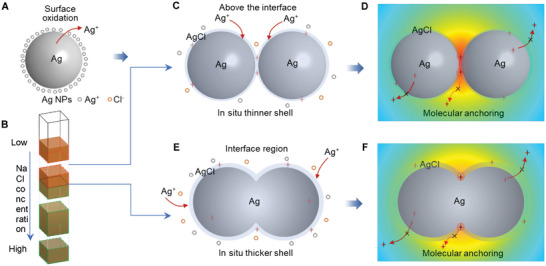
The proposed schematic model and physics nature of SM‐SERS. A) Ag NPs with released Ag^+^. The releasing of Ag^+^ occurs from the oxidation of metallic silver. B) Ag NPs colloid with interface in the presence of NaCl gradient. C,D) Ag NPs aggregates and electromagnetic field distribution at a low NaCl concentration above the interface. The released Ag^+^ from Ag NPs further reacts with Cl^−^, forming an in situ thinner AgCl packaging shell on the surface of the dimer. E,F) Ag NPs interconnection and fusion at interface region. A thicker AgCl shell forms on the surface of the aggregated particles.

Based on the previous literature, the dissolution and the releasing of Ag^+^ ions from the Ag NPs can possibly happen, e.g., by employing chloride (Cl^−^) for the oxidative etching of the Ag NPs,^[^ ^32^, ^33^ ^]^ or in the antimicrobial effect.^[^ ^34^, ^35^ ^]^ As the schematic illustration in **Figure** **5**, the oxidation of Ag NPs probably leads to the release of Ag^+^ ions from the Ag NPs and the final formation of precipitate of AgCl with Cl^−^ on the surface of the aggregated Ag NPs. The precipitated semiconductor shell of AgCl played a necessary condition in the discovery of the SM‐SERS discovery in 1997 and may remarkably enhance SERS properties.^[^ ^2^, ^3^ ^]^ The similar influence of adding NaCl on the morphology, structure, and SERS performance can also be confirmed in the electrochemical SERS system reported in 1974 (Figures S24 and S25, Supporting Information).^[^ ^1^ ^]^ The influence of Cl^−^ present in CV on SERS performance was further studied. The higher concentration of positively charged CV (10^−4^ or 10^−5^ m) can induce the aggregation of negatively charged Ag NPs thus producing SERS signal, even without the addition of NaCl.^[^ ^36^, ^37^ ^]^ When the concentration of CV decreases to 10^−8^ m, the SERS signal could not be obtained from the mixed solution of Ag NPs and CV, indicating that trace amounts of Cl^−^ present in CV would not affect SERS performance (Figure S26, Supporting Information). The presence of AgCl shell provides an additional contribution to SERS signals beyond the hotspots. By adding NaCl and AgNO_3_ to the Au colloid, an AgCl shell can also be formed on the surface of Au NPs. Compared to using only NaCl as an aggregation agent to generate hotspots in Au colloids, the in situ formation of AgCl shell on the surface of Au NPs can obviously decrease the LOD of CV from 10^−8^ to 10^−12^ m (Figure S27, Supporting Information).

The NaCl concentration gradient not only induces the different aggregation extent of Ag NPs but also controls the formation of AgCl shells with different thicknesses. Above the interface, Ag NPs show slight aggregation and interconnection, even the activation effect has worked by the thinner shell of AgCl (Figure 2A‐i and Figure 5C,D). Therefore, in this case, SERS displays only relatively weak signals. Below the interface, the over‐interconnected and fused structure, even the irregularly connected agglomerate, as well as the over‐precipitation of AgCl shell could deteriorate the SERS property again (Figure S28, Supporting Information). It is reasonable that the gradient‐distributed salt concentration is a key factor in the creation of the critical structure and activation state in the interface region of the Ag colloidal system. In the un‐interfaced solution obtained by adding excess NaCl to the mixture of Ag NPs and CV, the high concentration of NaCl leads to the serious aggregation of Ag NPs and the over‐precipitation of AgCl shell in the whole solution, resulting in the poor SERS performance (Figures S29–S31, Supporting Information). In addition, in the current study, different from the activation effect reported in SM‐SERS in 1997, the target molecule in the current salt‐gradient model has been added to the colloidal Ag NPs prior to the feeding of NaCl. Thus, during the precipitation of AgCl shell, the target molecule could be packaged into the AgCl activation shell (Figure 5; Figure S28, Supporting Information). This additional stabilization effect could contribute to an enhanced SERS sensitivity. Further investigation shows that other electrolytes, e.g., MgSO_4_, can also produce the same effect as the NaCl and highly improved SERS properties (Figure S32, Supporting Information). Therefore, excellent versatility and sensitivity are revealed in the current strategy.

## 3 Conclusion

In this work, a salt‐gradient model was proposed to accurately control the distribution of NaCl concentrations in one container from the top to the bottom of the solution. Thus, the influence of Cl^−^ concentrations on the morphology, structure, composition, and their evolution of Ag NPs hence on SERS properties have been systemically studied and optimized within one experimental setting. Different from the system of Au NPs, an obvious morphological and structural evolution, from individual spherical particle‐particle aggregation to interconnection, fusion, and even irregularly connected agglomerates, can be observed in the salt‐gradient model system of Ag NPs. The characterizations verified the composition evolution process on Ag NPs, along with the morphological and structural evolution. The above results indicate an unusual dissolution of Ag and the release of Ag^+^ ions from Ag NPs, to the final formation of AgCl which acts as the active and packaging shell to remarkably improve the SM‐SERS property. The current robust model system and SERS strategy, not only reveal a clear understanding of relevant processes, mechanisms, and physics nature in the famous SM‐SERS experiment for decades but also play an ideal substitution for current commercial solution‐based SERS protocols to significantly improve the SERS property. Therefore, we believe that the current model strategy with a unique interfacial platform offers a vast opportunity for cost‐effective, simple, fast, flexible, portable, and ultra‐trace SERS detection in a variety of fields.

## Conflict of Interest

The authors declare no conflict of interest.

## Author Contributions

L.L. synthesized the materials, observed the experimental phenomenon, and carried out the characterizations and performance. R.Z. and Y.G. carried out the experimental characterizations. H.Y. and Y.L. designed partial experiments, contributed to discussions and comments. J.G., S.L., and F.T. contributed to the SAXS characterization and analyzed the data. J.F. supervised the project, designed the experiments, and contributed in discussions, comments, and writing of the manuscript. All authors discussed the results.

## Supporting information



Supporting Information

## Data Availability

The data that support the findings of this study are available in the supplementary material of this article.
