# Supplementary material for: Updated Insights Into the Mechanism of Salt‐Induced Aggregation‐Based Single‐Molecule Surface‐Enhanced Raman Spectroscopy
Source: Adv Sci (Weinh). 2025 Mar 13;12(17):2417025. doi: 10.1002/advs.202417025 (PMC12061294; doi:10.1002/advs.202417025)
Supplement: Supplementary file 1 — Supporting Information [file ADVS-12-2417025-s001.docx]

Supplementary Information

**Updated Insights into the Mechanism of Salt-Induced Aggregation-Based Single-Molecule Surface-Enhanced Raman Spectroscopy**

Lingwei Li^1^, Ruiyuan Zhang^1^, Yu Guo^1^, Jiacheng Ge^2^, Si Lan^2^, Feng Tian^3^, Yi-Tao Long^4,*^, Hongjun You^1*^, Jixiang Fang^1*^

^1^Key Laboratory of Biomedical Information Engineering of Ministry of Education, School of Life Science and Technology, Xi’an Jiaotong University, Xi’an, Shaanxi, 710049, China

^2^Herbert Gleiter Institute of Nanoscience, School of Materials Science and Engineering

Nanjing University of Science &Technology, Nanjing, 210094, China

^3^Shanghai Synchrotron Radiation Facility, Shanghai Advanced Research Institute, Chinese Academy of Sciences, Shanghai, 201204, China

^4^[School of Chemistry and Chemical Engineering, State Key Laboratory of Analytical Chemistry for Life Science, Nanjing University, Nanjing, 210023, China](http://hysz.nju.edu.cn/ytlong/75/19/c37363a488729/page.htm)

*To whom correspondence may be addressed. E-mail: yitaolong@nju.edu.cn, hjyou@mail.xjtu.edu.cn, jxfang@mail.xjtu.edu.cn

**Materials and methods**

**Materials**

Silver nitrate (AgNO_3_), NaBH_4_, NaBr were purchased from China National Pharmaceutical Group Co., Ltd.; NaCl, CV, were purchased from Shanghai Aladdin Biochemical Technology Co., Ltd.; Sodium citrate (Na-Cit) was purchased from Tianjin Shengao Chemical Co., Ltd.. All chemicals were used without further purification. Milli-Q water (>18.0 MΩ cm) was purified with a Sartorius arium 611 UV ultrapure water system.

**Synthesis of Ag nanoparticles (NPs)**

The Ag NPs were synthesized by the stepwise seeding growth method based on the literature.^[1]^ A 20 mL of 1% Na-Cit solution and 75 mL of water were added to a beaker, and the mixture was heated to 70 ℃ for 15 min. After that, 1.7 mL of 1% AgNO_3_ solution was introduced to the mixture, followed by the quick addition of 2 mL of 0.1% freshly prepared NaBH_4_ solution. The reaction solution was kept stirring for 1 h and cooled to room temperature. The resulting Ag NPs were used as starter seeds. 2 mL of 1% Na-Cit solution was mixed with 75 mL of water and heated to boiling for 15 min. Next, 10 mL of Ag seeds solution was added while vigorously stirring, followed by the addition of 1.7 mL of 1% AgNO_3_ solution. After 30 min, 2 mL of 1% Na-Cit and 1.7 mL of 1% AgNO_3_ solution were added to the reaction solution, and reflux with vigorous stirring continued for another 30 min. After that, the reaction solution was cooled to room temperature. The Ag NPs with 25 nm were obtained. For the larger size of Ag NPs, the obtained 25 nm Ag NPs were used as seeds. A 2 mL of 1% Na-Cit was mixed with 80 mL of water and heated to boiling for 15 min. Next, 10 mL of the seed solution was added while vigorously stirring, followed by the addition of 1.7 mL of 1% AgNO_3_ solution. The reaction solution was kept vigorously stirring for 30 min and cooled to room temperature, and Cit-Ag NPs were obtained.

**Synthesis of Au nanoparticles (NPs)**

The Au NPs were synthesized based on a citrate reduction approach.^[2]^ For the first step, 100 mL of ultrapure water was added into a three-neck flat-bottom flask and heated to boiling. Then, 4 mL of 1 wt% Na-Cit solution was injected immediately, and 3.2 mL of 10 mM HAuCl_4_‧4H_2_O was added into it after 3 min. Subsequently, the reaction was kept boiling for 25 min and then cooled naturally, thereby obtaining the Au seeds. For the second step, 80 mL of ultrapure water and 20 mL of Au seeds were mixed in a three-neck flat-bottom flask and heated to boiling. Then, 2 mL of 1 wt% Na-Cit solution was injected immediately, and 0.2 mL of HAuCl_4_‧4H_2_O was added into the solution 3 min later. Then, an additional 0.2 mL × 9 dosage of HAuCl_4_ was injected in every 8 minutes. After the last precursor was added, the reaction was kept for 25 min, and Cit-Au NPs with the size of 25 nm were obtained. Replace the gold seed with 25 nm-Au NPs and repeat the second step, the Au NPs with the size of 50 nm can be obtained.

**Characterization**

The morphology of the Ag NPs was characterized using a transmission electron microscope (TEM, JEOL, JEM-2100 with an accelerating voltage of 200 kV). The *in situ* UV-Vis spectroscopy measurements were characterized using Ultraviolet-visible spectrometer (UV, Shimadzu, UV-60). UV spectra were obtained by collecting the signal from bottom to top of the solution after introducing the 100 µL of NaCl (1.5M) into the mixture of 500 µL Ag NPs and 2 mL analyte. The XPS data were obtained using an X-Ray Photoelectron Spectrometer (Thermo ESCALAB 250XI). Ag NPs at different positions were taken out and washed a couple of times and then dropped onto the silicon plate for characterization. The Michelson interferometer measurement was performed by a Michelson interferometer measurement based on the literature.^[3]^ The cell with solution was settled in one of the light paths vertically. A camera was used to take the pictures.

**Salt-gradient model**

The salt-gradient model was construct by adding small amount of NaCl in solution without any vibration. In detail, in a cuvette, 50 µL of 1.5 M NaCl was added in 2.5 mL of water, the NaCl aqueous was dropped in the bottom of water and then diffusion from bottom region until equilibrium. In order to characterize the distribution and diffusion process of NaCl in water, CV was used as a color tracer. Adding the mixture of 1.5 M NaCl and 10^-4^ M CV to water, without any vibration, the diffusion of mixture aqueous from bottom region of water can be observed.

**In situ Small Angle X-ray Scattering (SAXS)**

The SAXS was performed at the BL10U1 beamline of the Shanghai Synchrotron Radiation Facility (SSRF) using X-ray with a wavelength of 1.24 Å and distance of 27.6 m. A simple sample cell with thickness of 1 mm, width of 1cm, and height of 5 cm was prepared using polyimide film. 80 µL of Ag NPs, 320 µL of water, and 16 µL of 1.5 M NaCl were added to the sample cell. An interface is generated in the solution. The signals were collected along with the position from bottom to top of the solution. The NaCl aqueous solution with the same concentration was used as the background. In this study, we use the model of aggregated polydisperse spheres. In Guinier’s approximation that at low-q the scattering profile can be approximated as follows:

$$\text{I(Q)=}\text{N}_{\text{p}}{\text{n}_{\text{e}}}^{\text{2}}\text{e}^{\frac{\text{-}\text{q}^{\text{2}}{\text{R}_{\text{g}}}^{\text{2}}}{\text{3}}}$$

Where *N_p_* is the number of particles in X-ray illumination range, n_e_ is the number of electrons in one particle, *R_g_* is the overall radius of gyration. In this case, we consider that at low *Q* values, the scattering intensity is given by the Guinier law. *R_g_* and *I(Q)* can be determined by performing a linear fit to the Guinier plot: *ln(I)* vs. *Q^2^* (Figure S18), called the Guinier plot. If the aggregates are assumed to be spherical, their average radius is related to the obtained *R_g_* as follows:

$$\text{R}_{\text{g}}\text{=}\sqrt{\text{3}\text{α}}$$

Where *α* is the slope of the linear fit of *ln(I)* vs. *Q^2^* at low *Q* range.

The SAXS data has been processed by fit2d. The background has been corrected for each position by the equation:

$\text{I(Q)=}\frac{\text{I}_{\text{sample}}}{\text{T}_{\text{sample}}}\text{-}\frac{\text{I}_{\text{bg}}}{\text{T}_{\text{bg}}}$

Where *I_sample_* and *I_bg_* are the scattering intensity of the sample and the background, and *T_sample_* and *T_bg_* are the transmissions of the sample and the background.

The size distribution curves of nanoparticles at different detection positions (Figure S19) were obtained by:

$$\text{I(Q)=}\frac{\text{S}}{\text{V}_{\text{p}}}{\text{[3}\text{V}_{\text{p}}\text{(}\text{ρ}_{\text{p}}\text{-}\text{ρ}_{\text{m}}\text{)}\text{Φ}\text{ (QR)]}}^{\text{2}}\text{+}\text{bkg}$$

$\text{Φ}\text{(x)=(}\text{sinx-xcosx}\text{)/}$x^3^

Where *(ρ_p_-ρ_m_)* is the contrast between the particle and the matrix, *R* is the radius, *V* is the volume, *Φ(x)* is a function that peaks at *Q* = 0 and falls monotonously at small *Q* values, *b_kg_* is the background.

**SERS spectroscopy**

The SERS spectra measurements were performed in a confocal microscope-based Raman spectrometer (B&W Tek Inc., i-Raman Plus) with a 785 nm laser. The detection was based on the reflection Raman through a laser probe, which receives scattered light with the same probe after irradiating the solution. SERS spectra were obtained by collecting the signal of the solution after introducing the different volumes of NaCl (1.5 M) into the mixture of 500 µL Ag NPs or Au NPs and 2 mL analyte, and the acquisition time was 20 s.

**The calculation of single molecule**

The molecular number in probe volume can be calculated based on the literature.^[4]^ The probe volume can be calculated through the equation:

$$\text{V=π}\text{r}^{\text{2}}\text{h}$$

where *r* is the radius of the laser spot which is ~40 µm, *h* is estimated from depth-of-field considerations, ~0.6 mm.

According to the above equation, the probe volume can be calculated as shown in the following:

$$\text{V=π}\text{r}^{\text{2}}\text{h=π}{\text{(}\text{4}\text{×}\text{10}^{\text{-5}}\text{ m)}}^{\text{2}}\text{×}\text{6}\text{×}{10}^{-4}\text{ m=}\text{3.0}\text{×}\text{10}^{\text{-12}}\text{m}^{\text{3}}=3.0\times{10}^{-9}L$$

The number of molecules in the probed volume can be calculated by the equation:

$$\text{N=V}\text{×}\text{C}\text{×}N_{A}$$

where *V* is probe volume, *C* is the concentration of CV solution in the cuvette, *N_A_* is the Avogadro's constant. The target solution contains 2 mL of CV (10^-15^ M), 500 µL of Ag colloid and 100 µL of NaCl, which give the final concentration of CV is 0.77×10^-15^ M in the solution.

According to the above equation, the N are estimated as following:

$$\text{N=V×C×}N_{A}\text{=3.0×}\text{10}^{\text{-9}}\text{L}\text{×0.77×}\text{10}^{\text{-15}}\text{M×6.02×}\text{10}^{\text{23}}\text{=}\text{1}\text{.4}$$

The molecule number in the probe volume reaches to single molecule level.

**Raman spectroscopy on electrode**

The electrochemical cell was designed as previous literature.^[5]^ Firstly, the ITO electrode was electrochemically deposited Ag in a 10 mM AgNO_3_ solution at a deposition voltage of 5V and a deposition time of 15 min. The ITO/Ag (1 cm×2 cm) electrode, copper sheet and saturated calomel were set as the working electrode, the subsidiary electrode, and the reference electrode (S.C.E.). The solution in contact with the ITO/Ag electrode during the experiments was 0.5 M NaCl or NaNO_3_ aqueous containing different concentration of analytical grade pyridine. The electrode was then subjected in the cell to cyclic linear potential sweeping for about 15 min at 0.5 V/s between +200 mV and -300 mV relative to the saturated calomel potential. After the electrochemical experiments, the electrode was washed three times using deionized water. The spectra of electrode were recorded using the confocal microscope-based Raman spectrometer with a laser of 785 nm wavelength and 15 mW power.


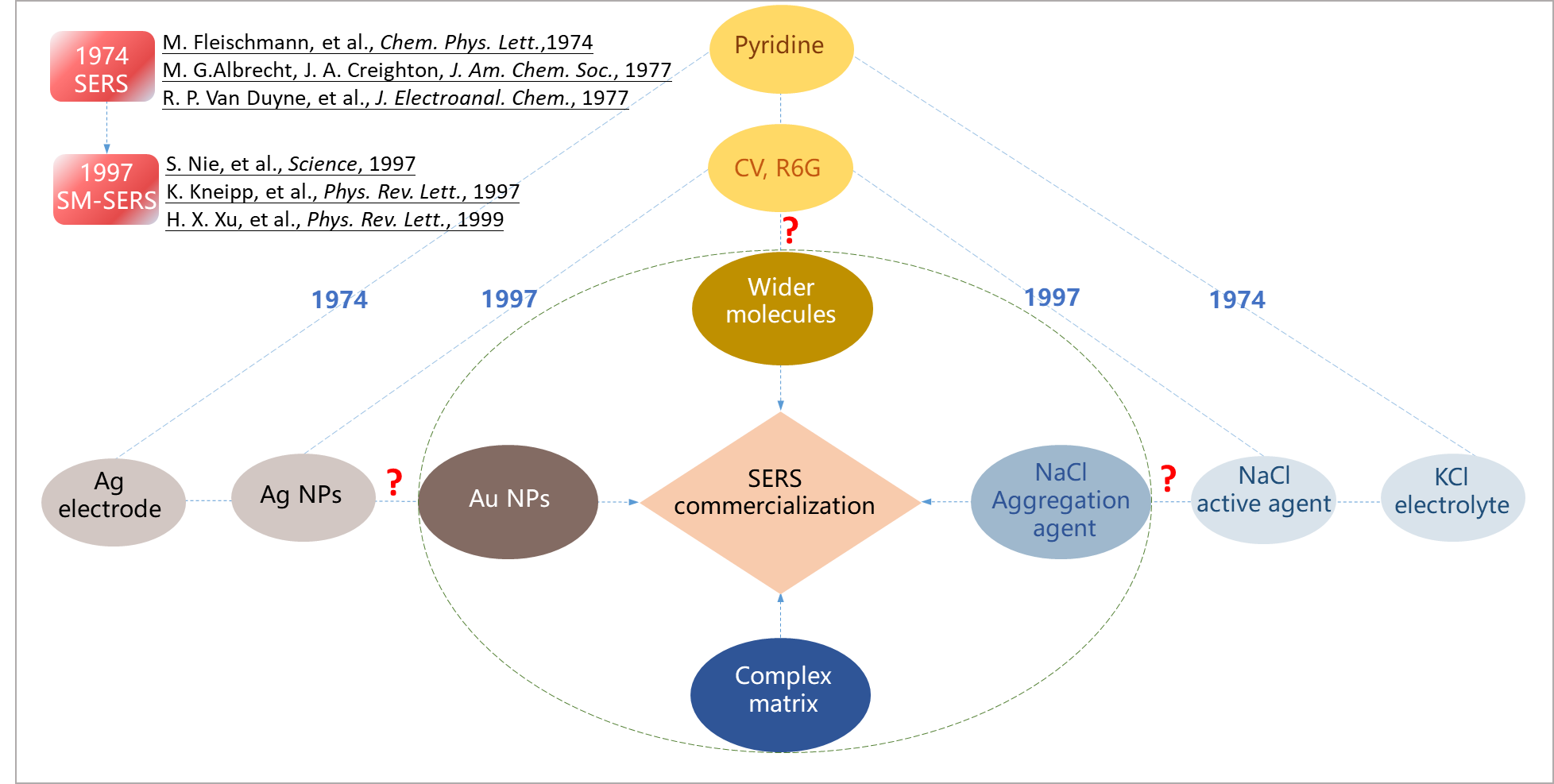


**Figure S1.** The common features of SERS and SM-SERS at early studies.


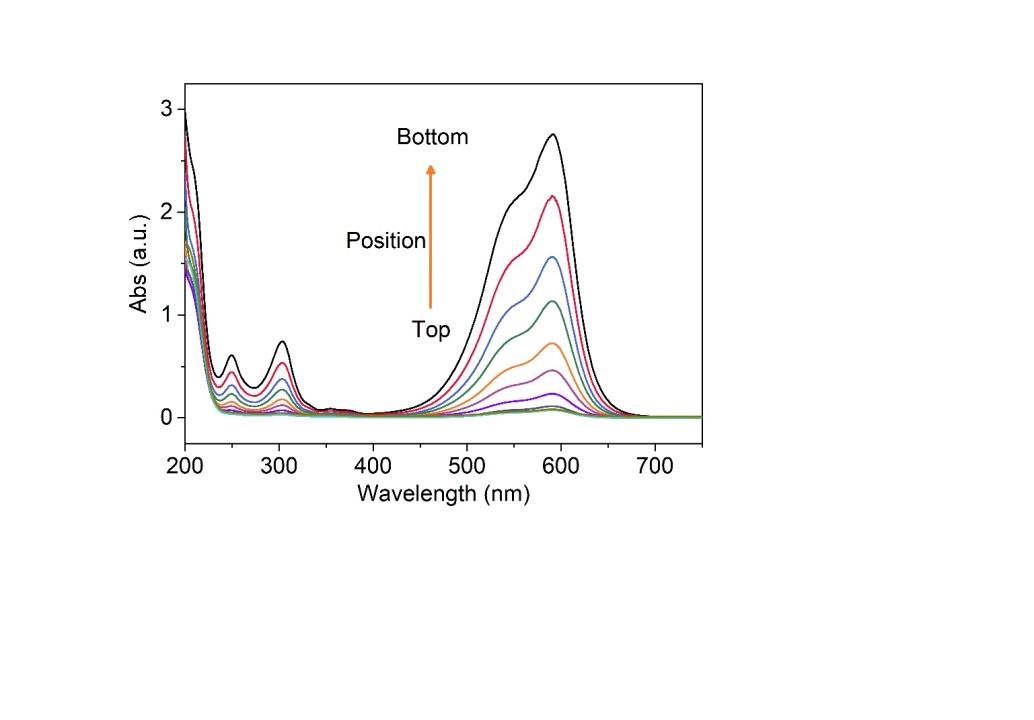

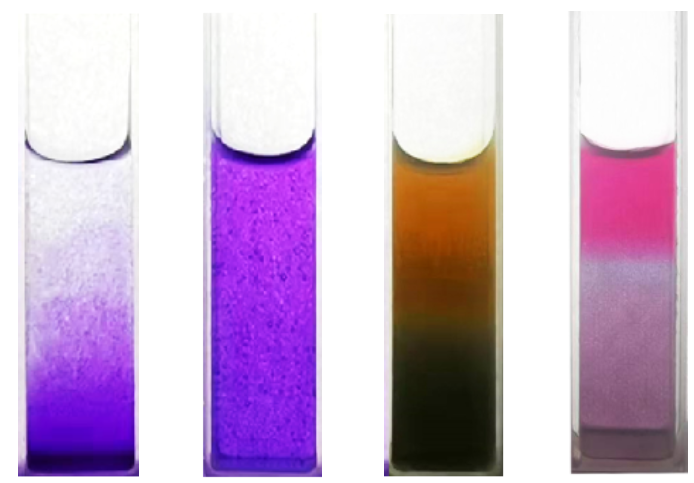


E

A

B

C

D

**Figure S2.** The salt-gradient model system solution. A) The mixture of CV and NaCl was dropped into water and kept stationary, an interface was shown in the solution. B) A few drops of CV solution added to water and diffused in the whole solution. C) A few drops of NaCl added to Ag colloid and kept stationary. A liquid-liquid interface was formed. D) A few drops of NaCl added in Au colloid and kept stationary. A liquid-liquid interface was formed. E) The position-dependent UV-Vis spectra for a NaCl and CV mixing solution as shown in figure A.


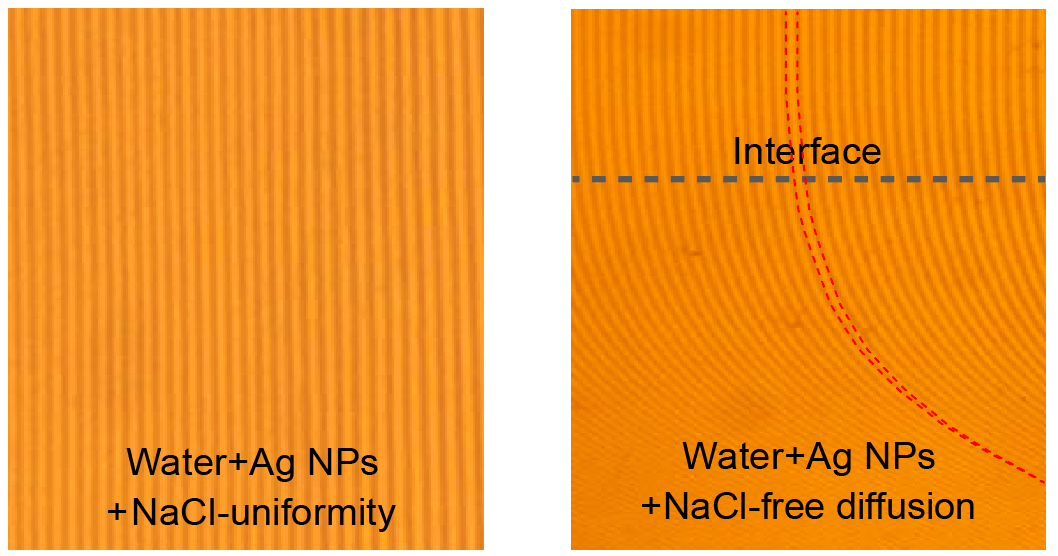


A

B

**Figure S3.** A) Image of interference fringes obtained from Michelson interferometer measurement for a specimen of NaCl dropping into colloidal Ag NPs, then shaking. B) Image of interference fringes obtained from Michelson interferometer measurement for a solution with interface by means of NaCl dropping into colloidal Ag NPs, then without any vibration.


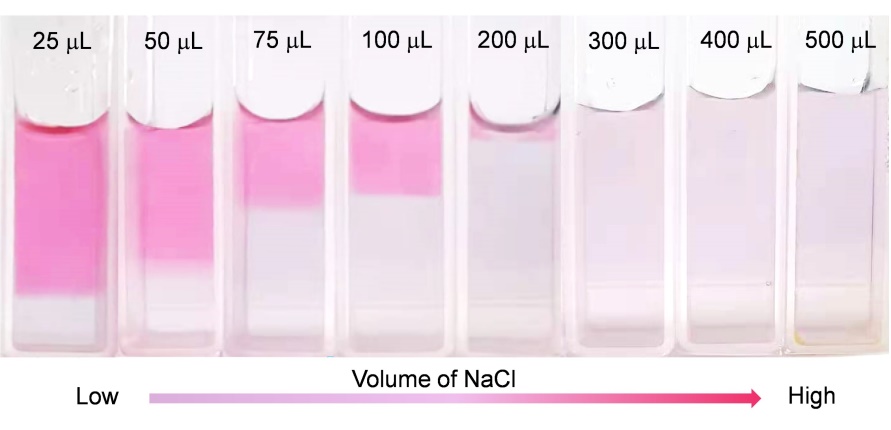


**Figure S4.** The Au NPs colloid with different adding volumes of NaCl solution.


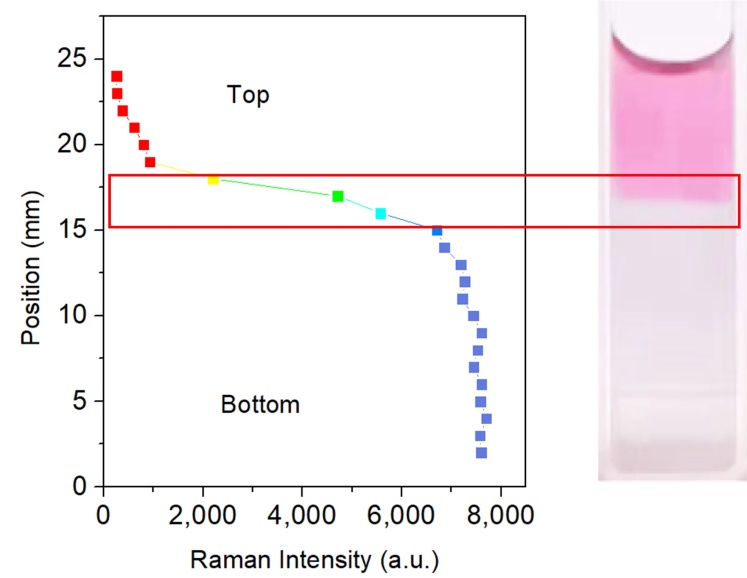


**Figure S5.** The SERS intensity of CV molecule at 1173 cm^-1^ changed as a function of detection position in Au colloid.


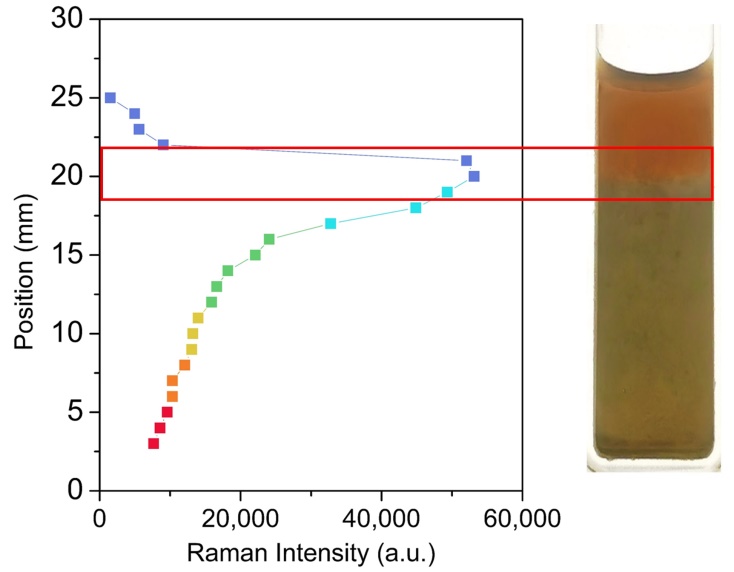


**Figure S6.** The SERS intensity of CV molecule at 1173 cm^-1^ changed as a function of detection position in Ag colloid.


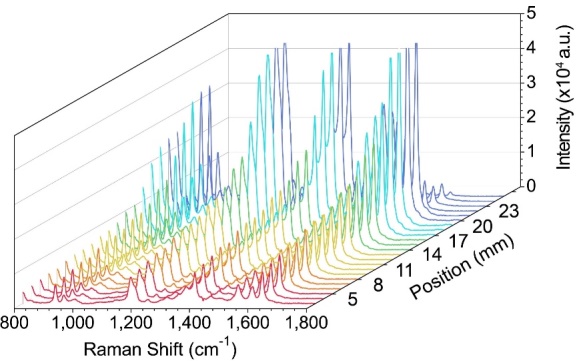

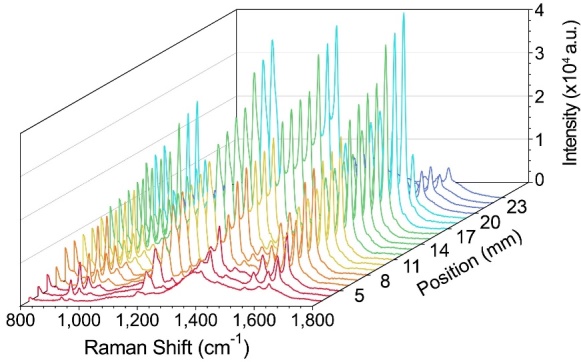

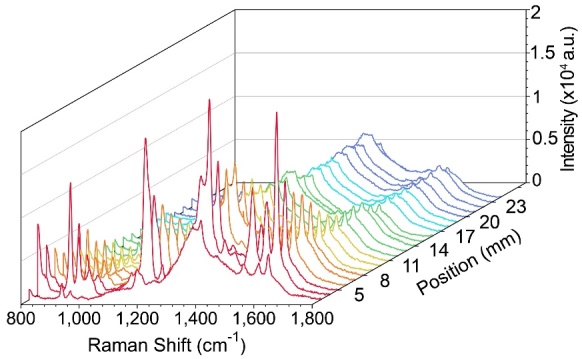

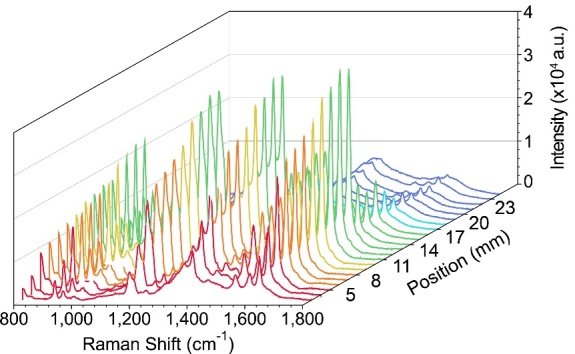


A

B

C

D

**Figure S7.** The position-dependent SERS spectra of 10^-8^ M CV. The SERS spectra were collected at the different positions of the solution, in which A) 25 µL, B) 50 µL, C) 75 µL, and D) 100 µL of NaCl were added in the mixture of 500 µL Ag NPs and 2 mL CV solution without shaking.


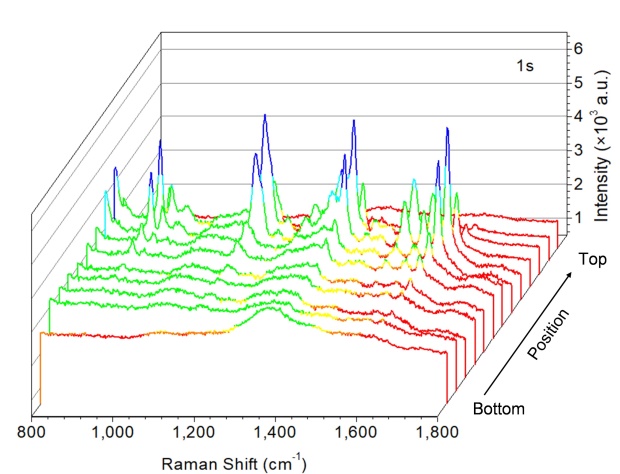

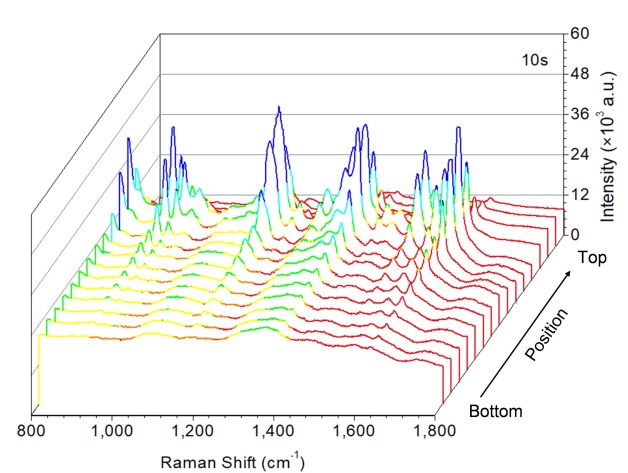


A

B

**Figure S8.** The position-dependent SERS spectra of CV (10^-8^M) with shorter integration time: A) 10s and B) 1s.


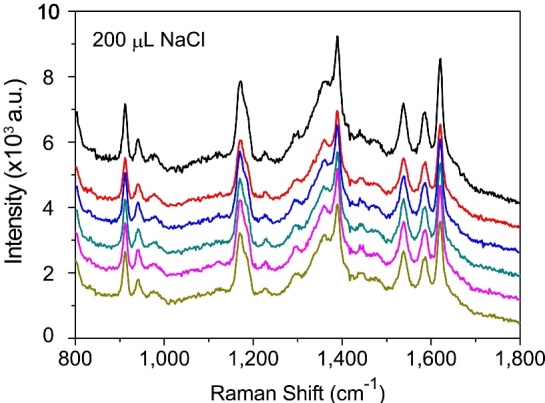

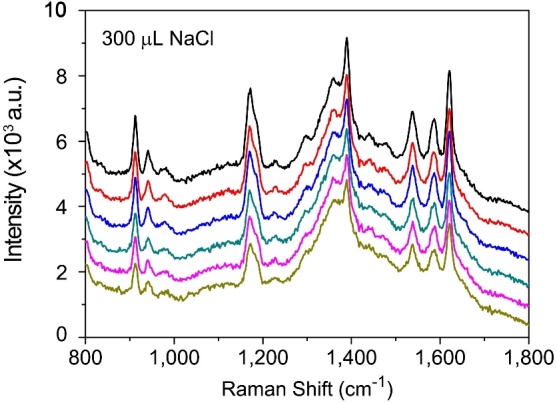

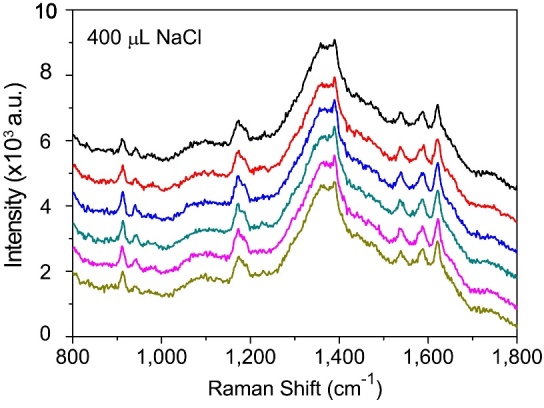

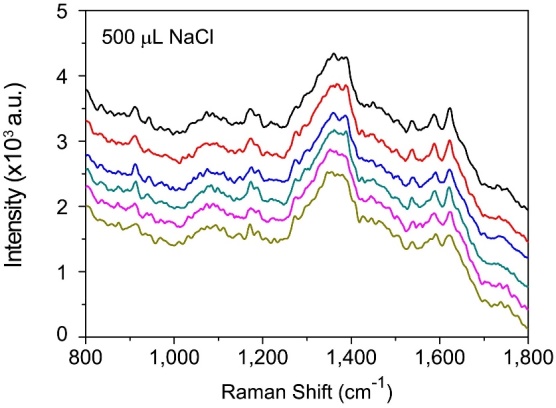


A

C

B

D

**Figure S9.** The SERS spectra of 10^-8^ M CV in the un-interface solution. The SERS spectra were collected in the un-interface solution, in which A) 200 µL, B) 300 µL, C) 400 µL, and D) 500 µL of NaCl were added in the mixture of 500 µL Ag NPs and 2 mL CV solution.


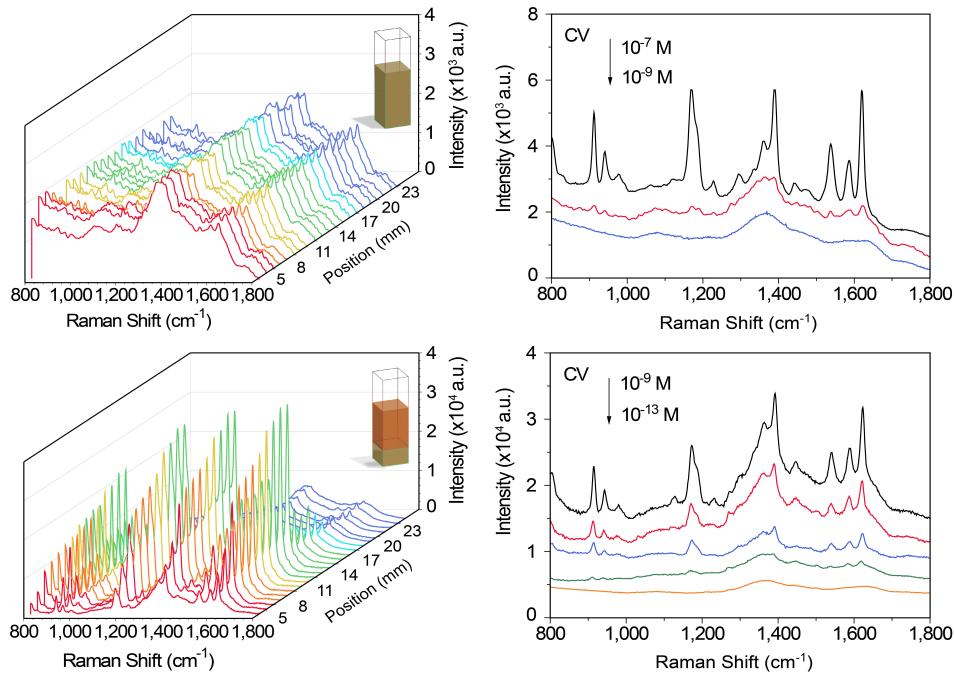


A

B

C

D

**Figure S10.** SERS detection of CV with different amounts of NaCl. A) Position-dependent SERS spectra of 10^-8^ M CV in the case of feeding volume of 500 µL NaCl into the mixture of 500 µL Ag NPs and 2 mL CV. B) SERS spectra at different concentrations of CV molecules with the feeding of 500 µL NaCl. C) Position-dependent SERS spectra of 10^-8^ M CV with 50 µL of NaCl. D) SERS spectra at different concentrations of CV molecules with the feeding of 50 µL NaCl.


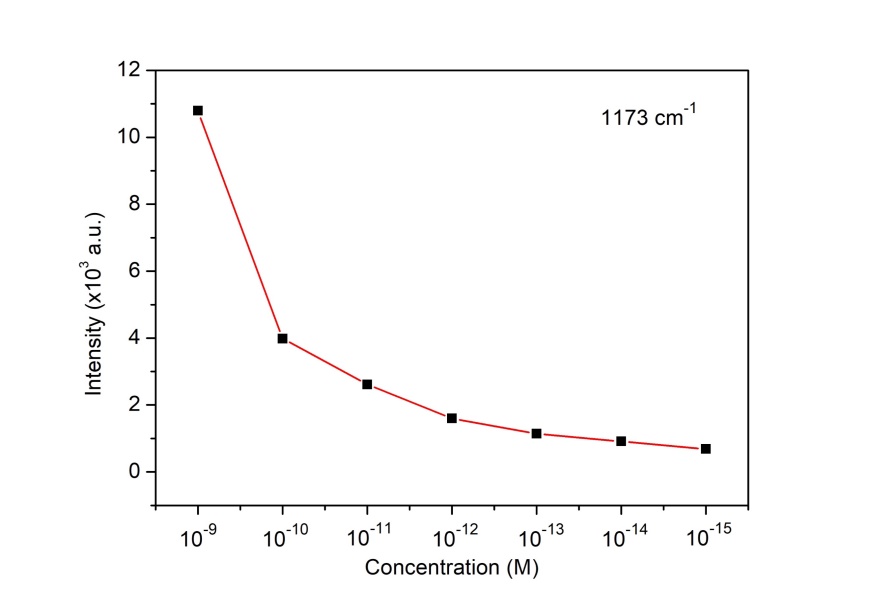


**Figure S11.** The SERS intensity of CV at 1173 cm^-1^ as a function of CV concentration in Ag colloid.


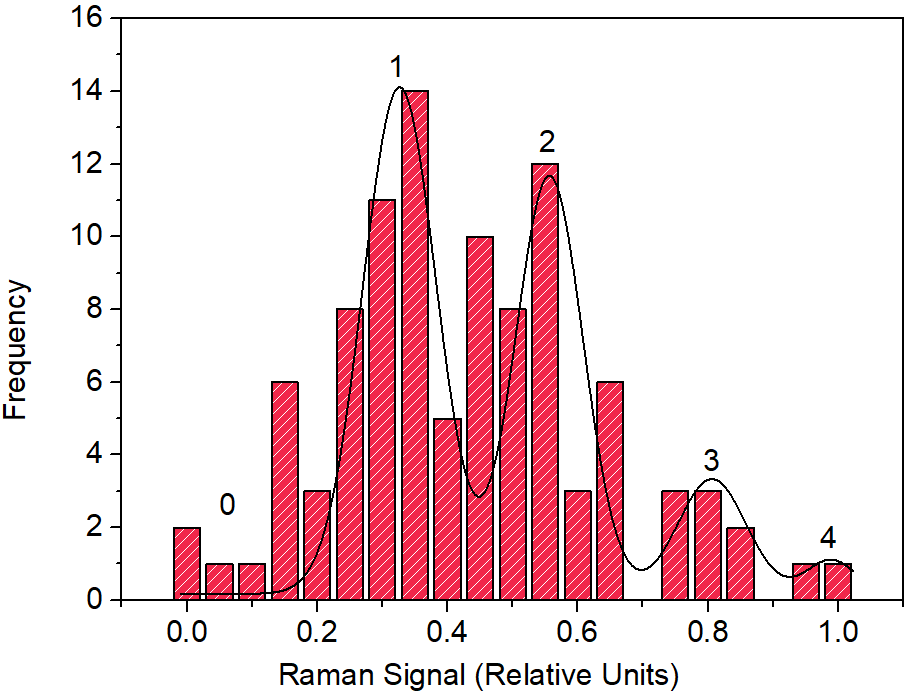

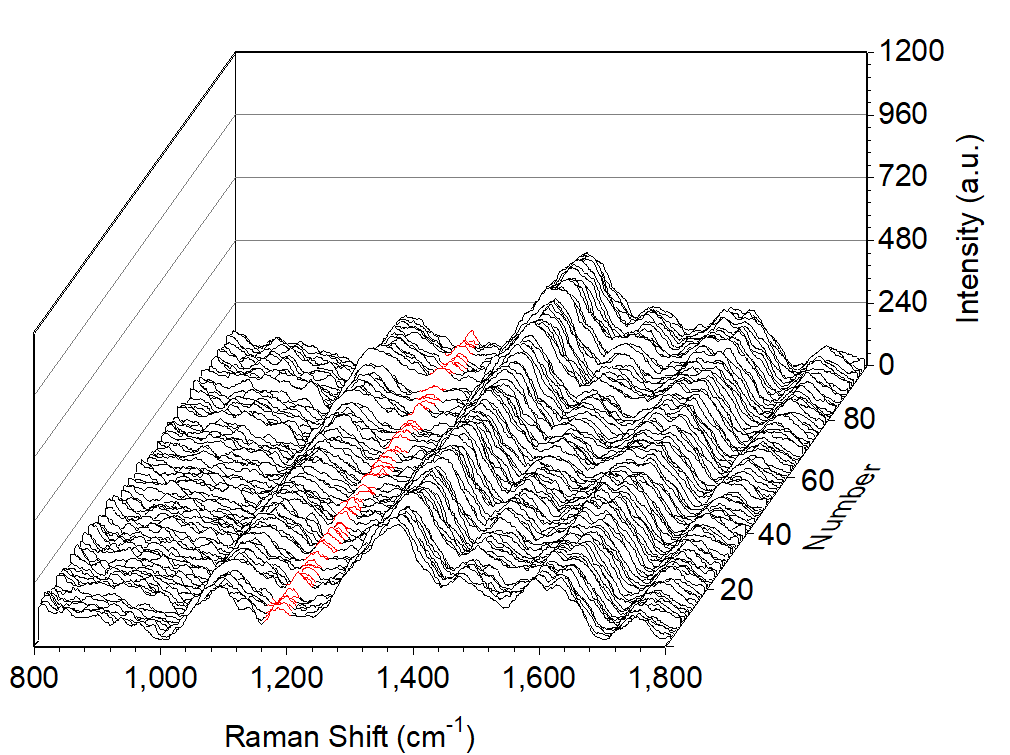


A

B

**Figure S12** A) 100 SERS spectra collected for10^-15^ M CV molecules in the probed volume. Each spectrum was acquired in 2 seconds. B) Statistical analysis of 100 SERS measurements (1173 cm^-1^ Raman line) for10^-15^ M CV molecules in the probed volume.


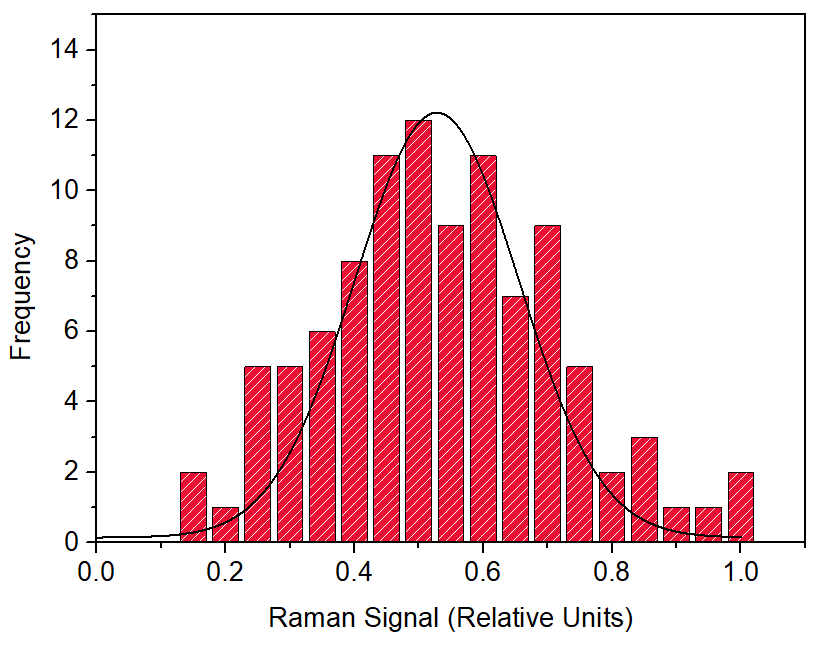

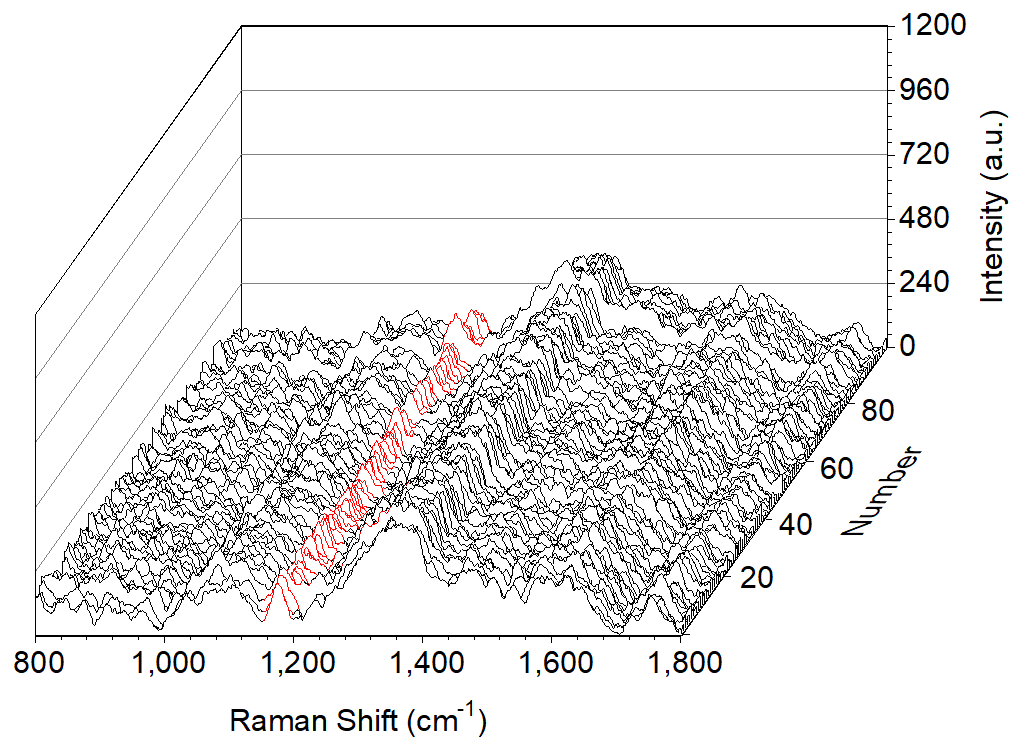


A

B

**Figure S13** B) 100 SERS spectra collected for10^-14^ M CV molecules in the probed volume. Each spectrum was acquired in 2 seconds. A) Statistical analysis of 100 SERS measurements (1173 cm^-1^ Raman line) for10^-14^ M CV molecules in the probed volume.


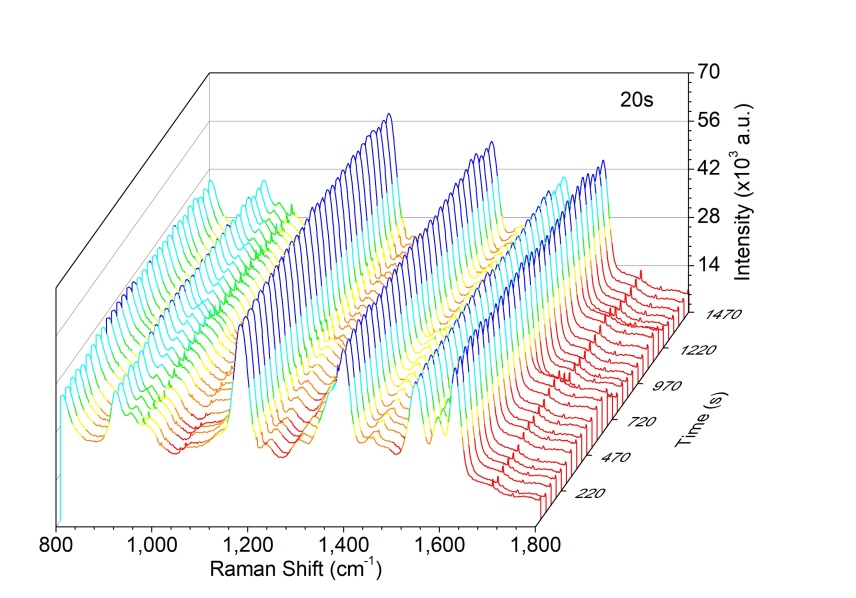


**Figure S14.** The time-dependent SERS spectra of 10^-8^ M CV at interface region.


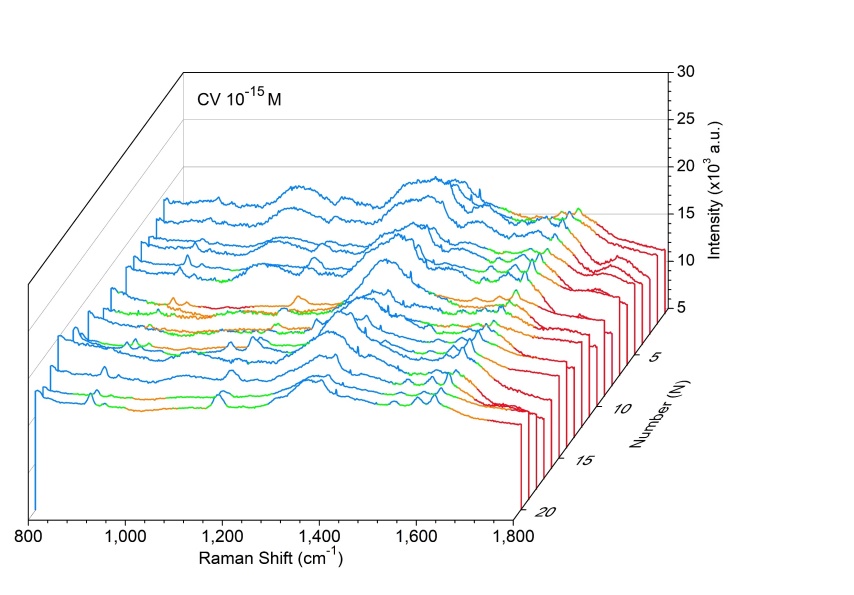


**Figure S15.** SERS spectra of 10^-15^ M CV detected in 20 batches.


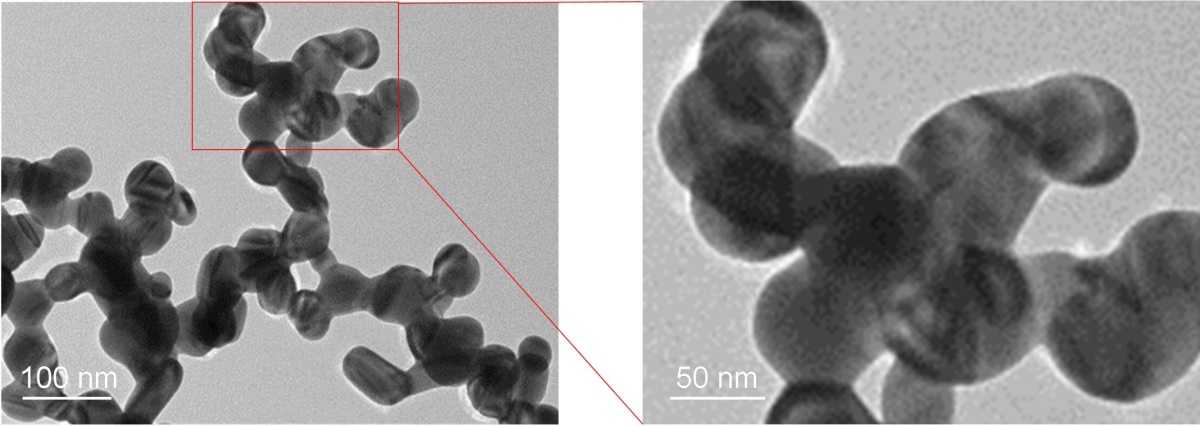


A

B

**Figure S16.** A) The TEM image of Ag NPs at interface region. B) The partial enlarged view of connected Ag NPs.


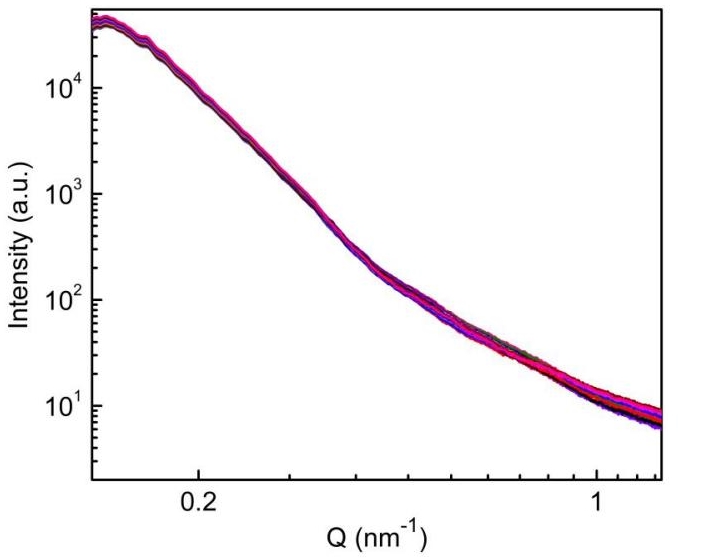


**Figure S17.** 1D SAXS scattering profiles at different positions of Ag NPs colloid with interface.


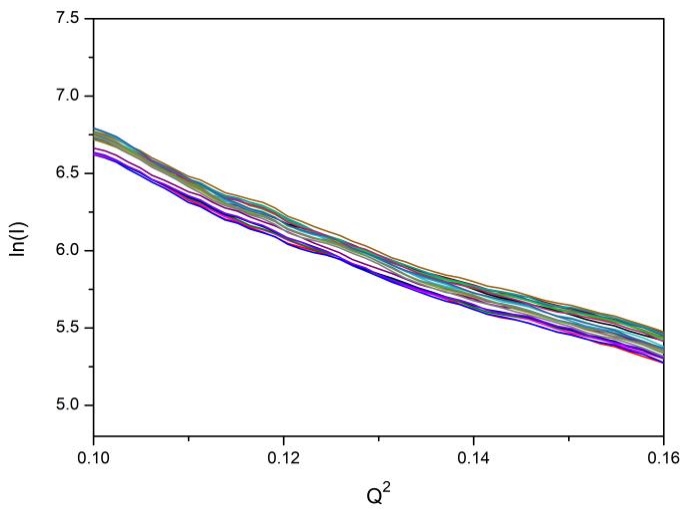


**Figure S18.** The linear fit of ln(I(Q)) vs. Q^2^_._


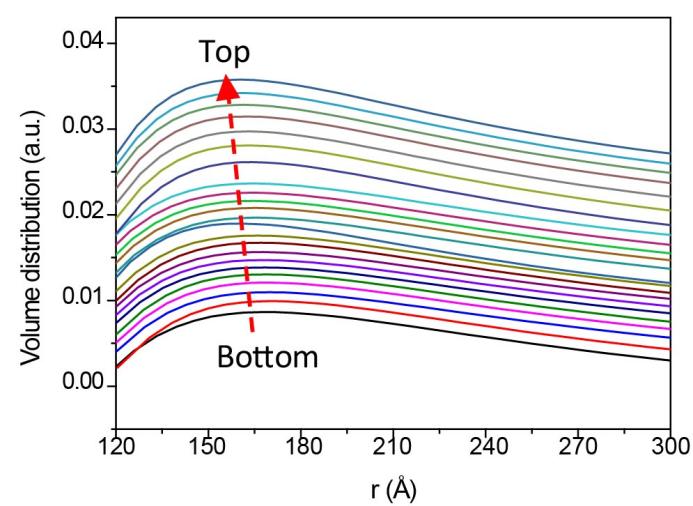


**Figure S19.** The size distribution curves of nanoparticles at different detection positions.


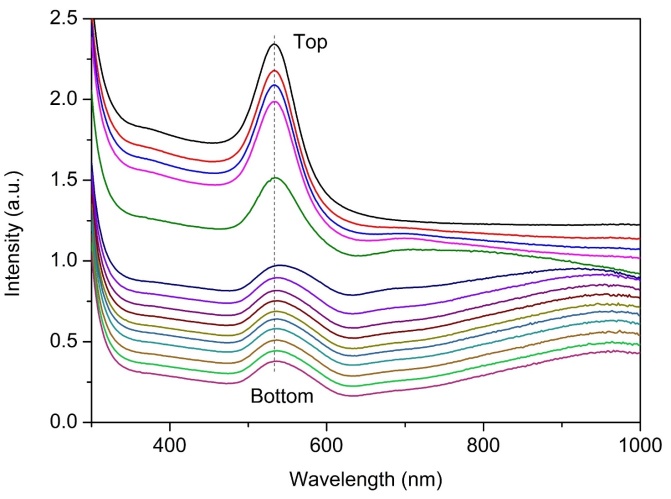

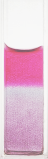


**Figure S20.** The position-dependent UV spectra of Au NPs colloid with interface by adding small amount of NaCl. 100 µL of 1.5 M NaCl was added into 2 mL of Au NPs colloid. The UV spectra were collected at different positions.


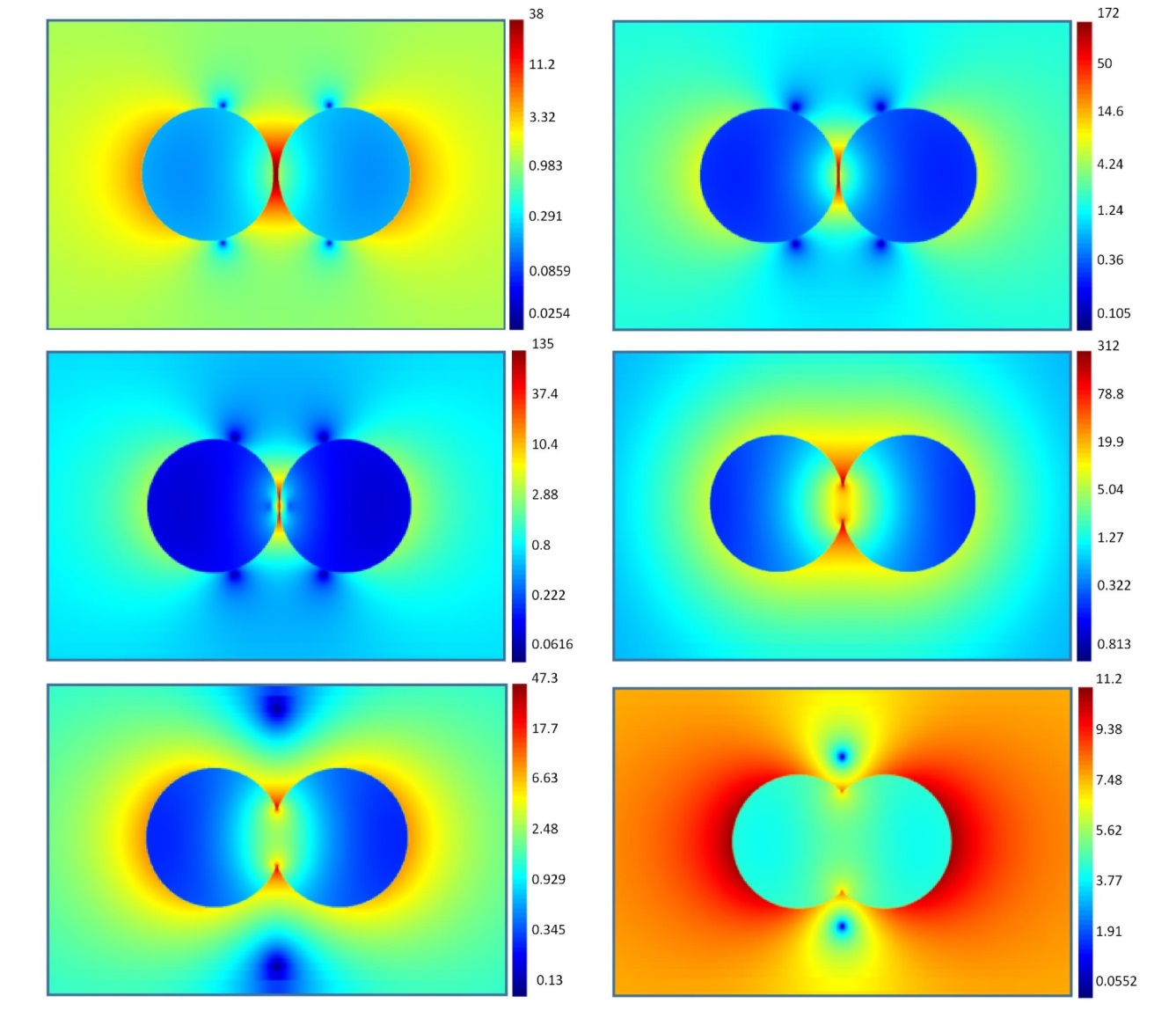


A

B

C

D

E

F

**Figure S21.** Electric field distribution of different aggregation states of Ag NPs using FDTD models under 785 nm laser excitation. The distance between Ag NPs were set as A) 2 nm, B) 0.5 nm, C) 0 nm, D) -1 nm, E) -5 nm, F) -10 nm, respectively.


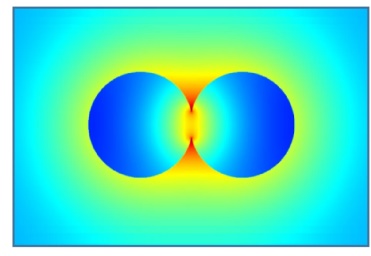


0.81

0.32

1.27

5.04

78.8

312


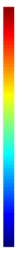


19.9

a

b


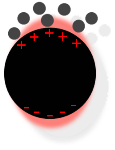


、

NaCl

Crystal

violet


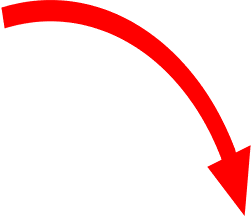

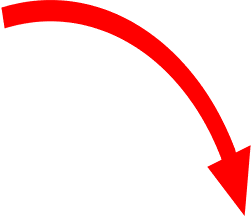

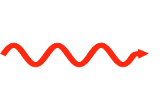

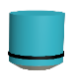


Laser


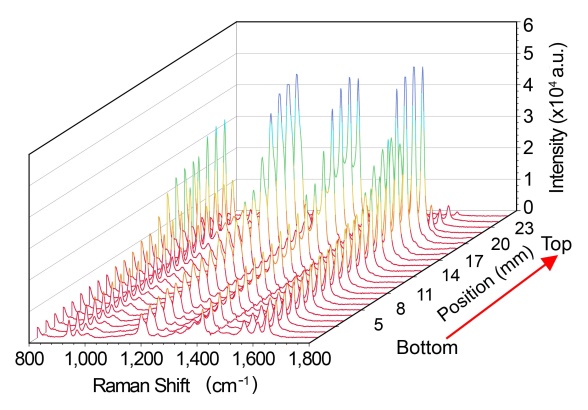


c


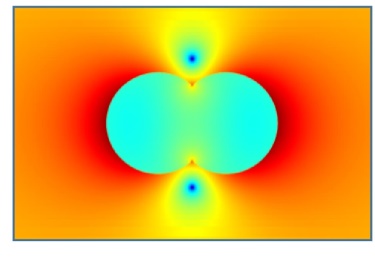

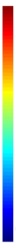


0.06

1.91

3.77

5.62

9.38

11.2

7.48

d

A

B

C

D

**Figure S22.** A) Schematic diagram of SERS detection at the delaminated interface of Ag NPs colloid. B) Electric field distribution of Ag NPs above the interface. C) Electric field distribution of Ag NPs at the interface region. D) Position-dependent SERS spectra of 10^-8^ M CV in the delaminated solution with 100 µL of 1.5 M NaCl.


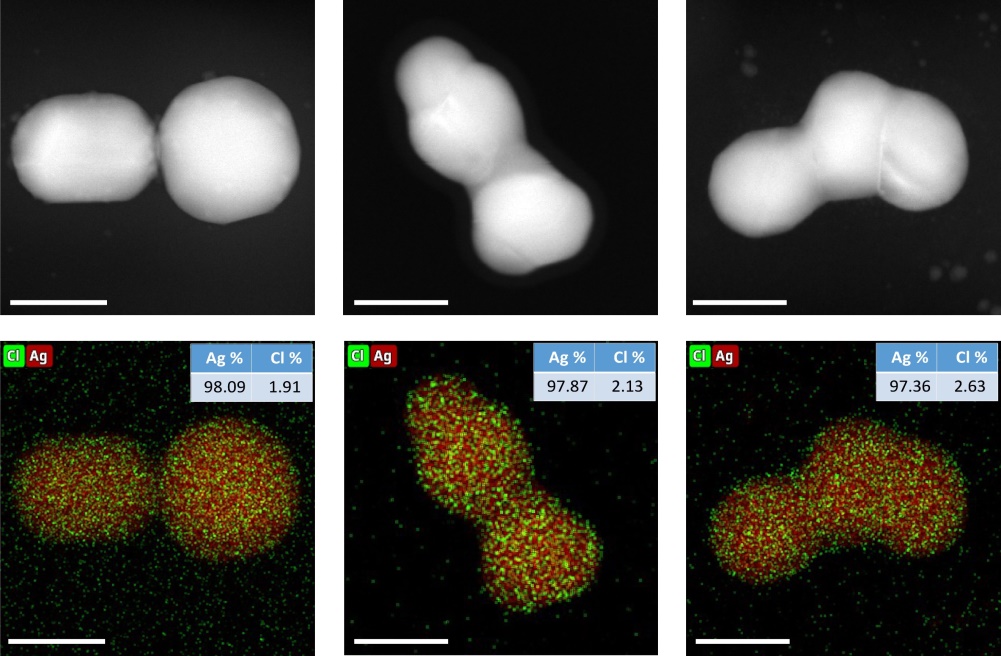


A

B

C

**Figure S23.** The EDS element mapping of aggregated Ag NPs at different positions of solution with interface. A) Ag NPs above the interface, B) Ag NPs at the interface region, C) Ag NPs below the interface. NaCl was added into the Ag NPs colloid to introduce inhomogeneous aggregation of Ag NPs. Ag NPs at the different positions were taken out and washed a couple of times to remove excess NaCl, then EDS characterization was performed. The scale bars are 50 nm.


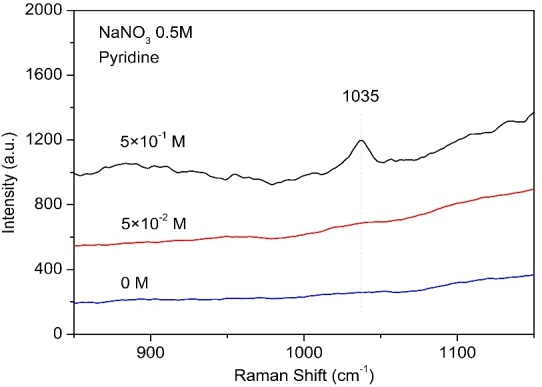

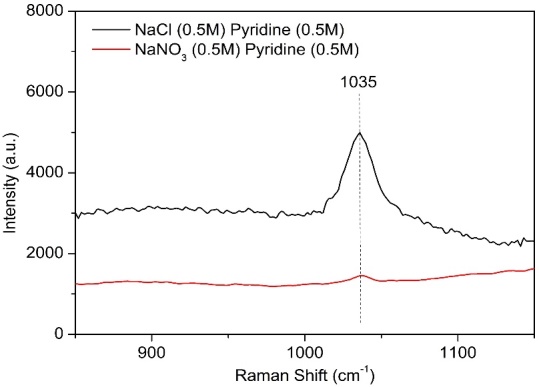

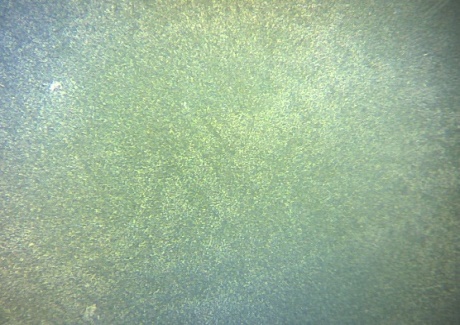

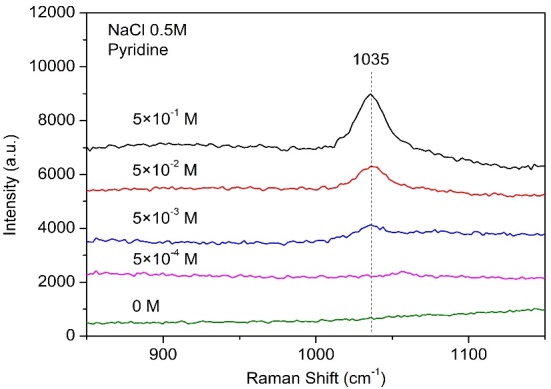


A

B

C

D

**Figure S24.** The effect of NaCl and NaNO_3_ on the SERS prosperity of pyridine in electrode system. A) The optical photograph of Ag NPs deposited on Ag electrode. B) SERS spectra of 0.5 M pyridine by using 0.5 M of NaCl and NaNO_3_ as electrolytes. C) SERS spectra of pyridine at different concentrations with 0.5 M of NaCl. D) SERS spectra of pyridine at different concentrations with 0.5 M of NaNO_3_.


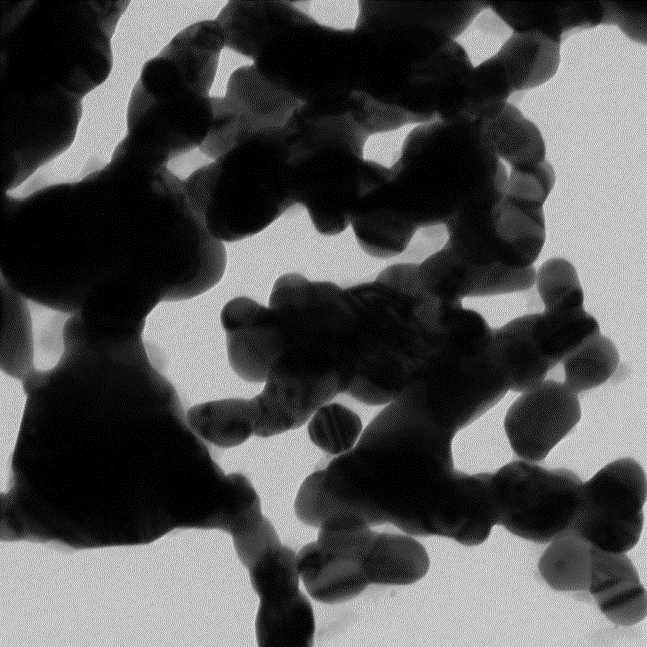


200 nm


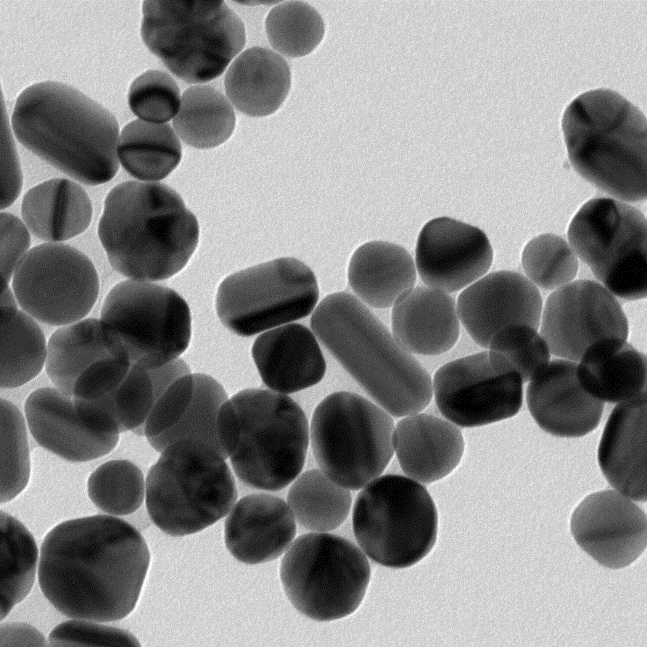


100 nm

A

B

**Figure S25.** TEM images of Ag NPs after the addition of NaCl and NaNO_3_. A) The interconnection and fusion morphology of Ag NPs after addition of 100 mM NaCl in Ag colloid. B) The individual Ag NPs after addition of 100 mM NaNO_3_ in Ag colloid. No interconnection and fusion of the Ag NPs occurred with the addition of NaNO_3_ because no precipitate shell was formed.


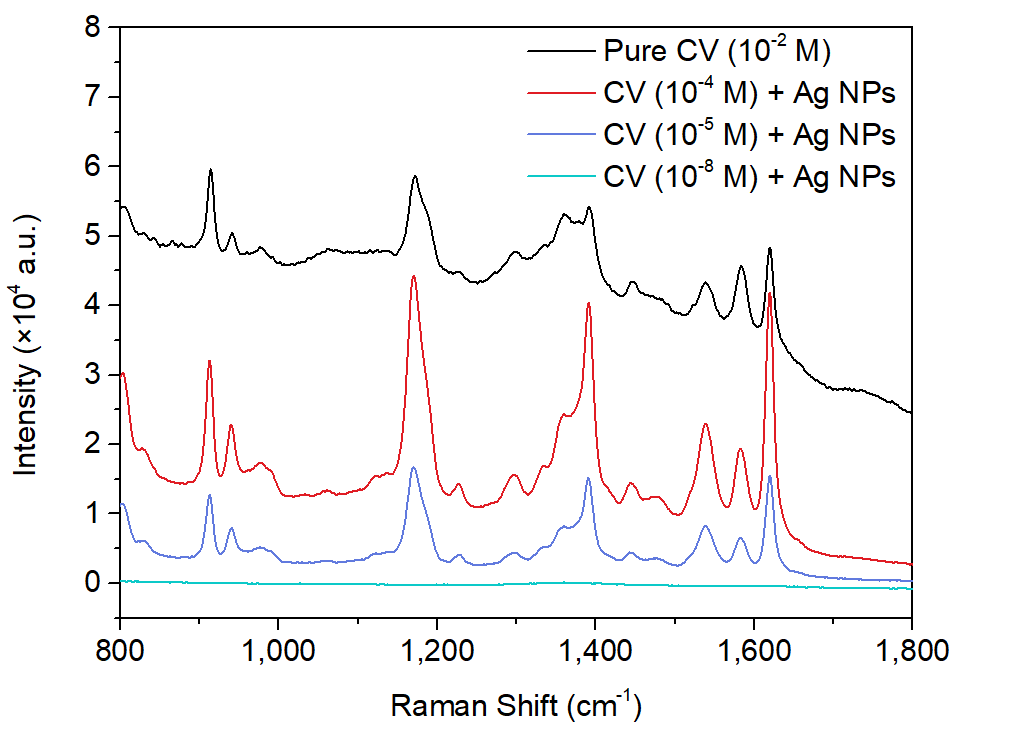


**Figure S26.** The effect of CV concentration on the SERS property.


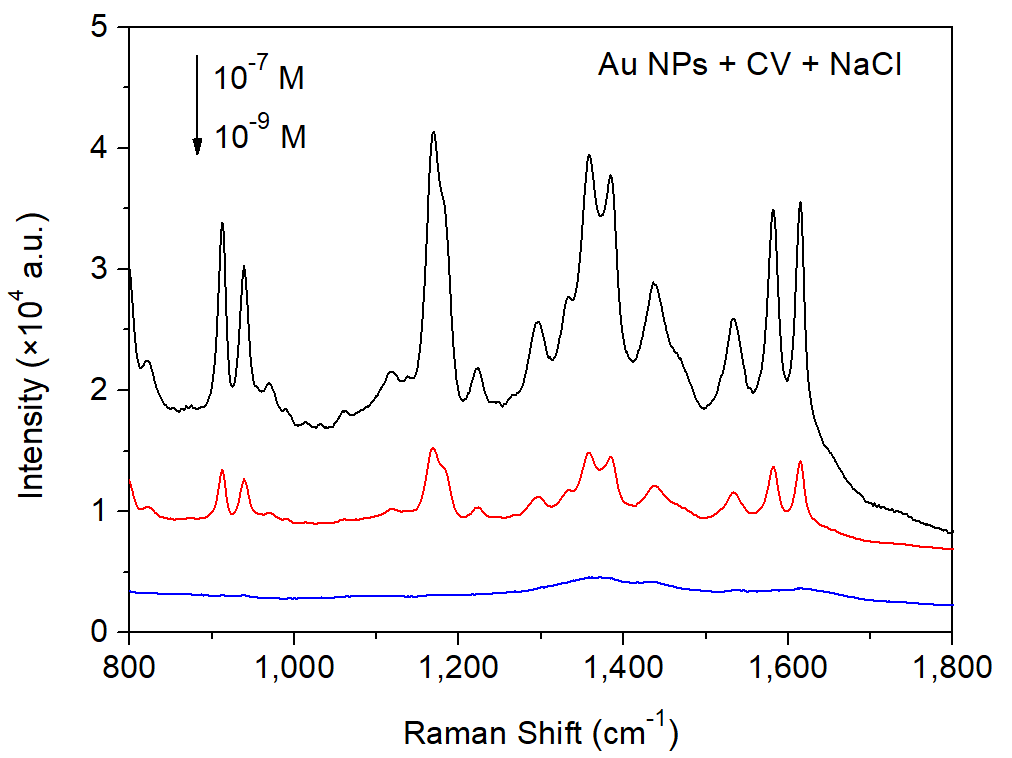

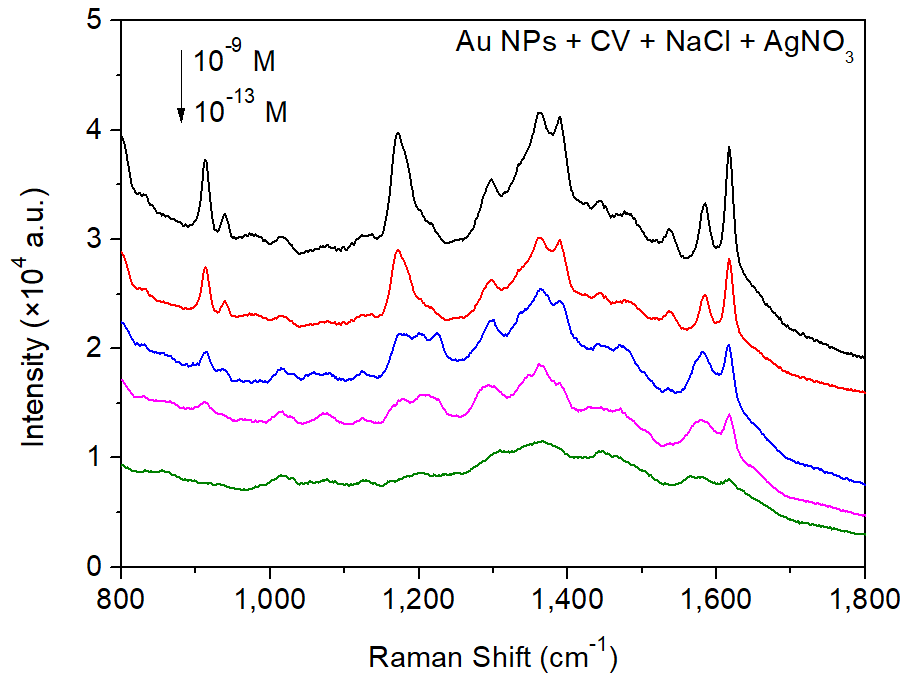


A

B

**Figure S27.** A) The SERS spectra of CV in Au colloid with NaCl as aggregation agent. B) The SERS spectra of CV in Au colloid with AgCl shell.


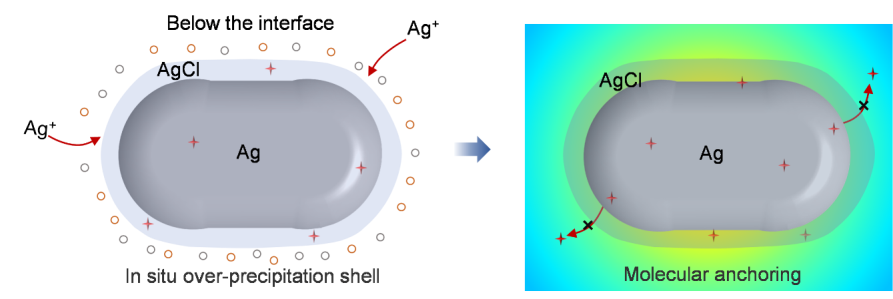

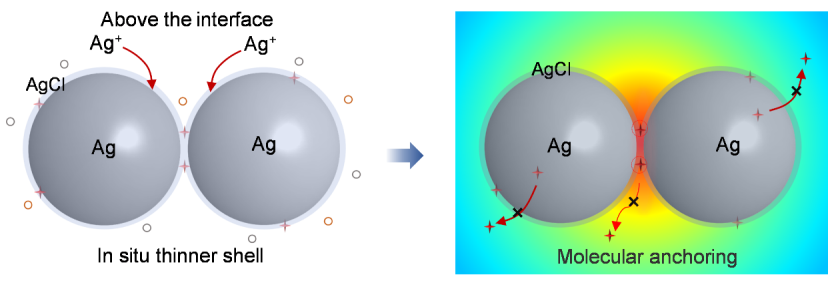

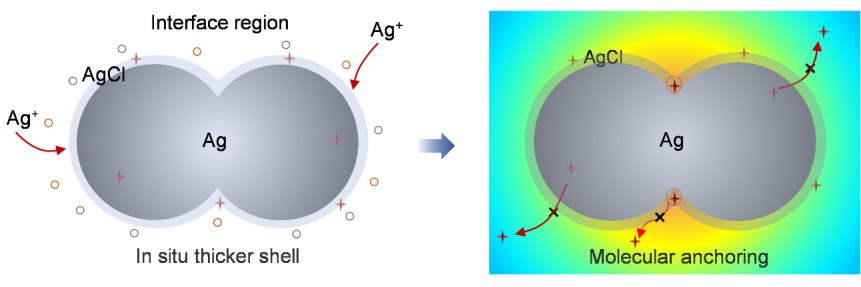

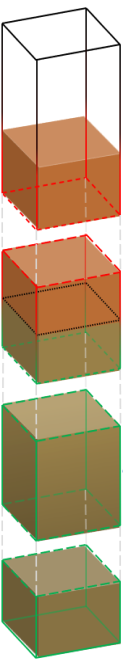


NaCl concentration

Low

High

A

B

C

E

D

F


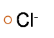

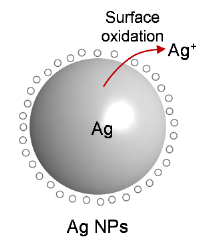

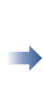

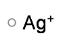


G

H

**Figure S28.** The proposed schematic model and physics nature of SM-SERS. A) Ag NPs with released Ag^+^. The releasing of Ag^+^ occurs from the oxidation of metallic silver by dissolved oxygen and protons. B) Ag NPs colloid with interface in the presence of NaCl gradient. C-D) Ag NPs aggregates and electromagnetic field distribution at a low NaCl concentration above the interface. The released Ag^+^ from Ag NPs further reacts with Cl^-^, forming an in-situ thinner AgCl packaging shell on the surface of the dimer. E-F) Ag NPs interconnection and fusion at interface region. A thicker AgCl shell forms on the surface of the aggregated particles. G-H) Ag NPs with serious interconnection and fusion at a high NaCl concentration. An over-precipitation AgCl shell formation on the surface of the fused particles. The loss of hot spots due to interconnection and fusion of Ag NPs resulting in a weak electromagnetic field.


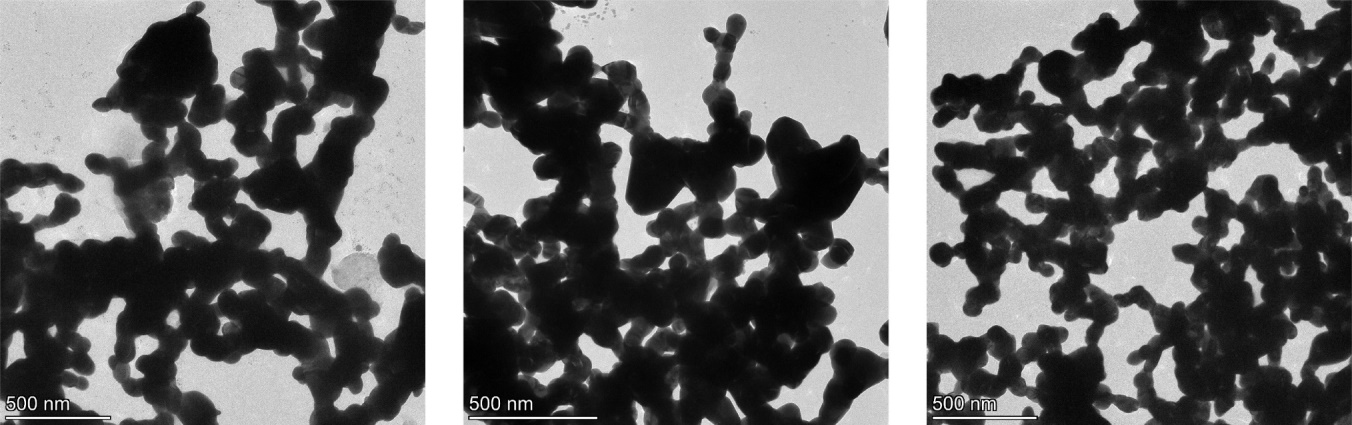


A

B

C

**Figure S29.** The TEM images of Ag NPs at different positions of solution with excess of NaCl (500 µL). 500 µL of NaCl was added into the Ag NPs colloid and diffused to the whole solution. The aggregation of Ag NPs occurred in the whole solution under excess NaCl and no interface formed. The Ag NPs at different positions were taken out and washed a couple of times to remove excess NaCl for TEM characterization. A) the top of the solution, B) the middle of the solution, and C) the bottom of the solution.


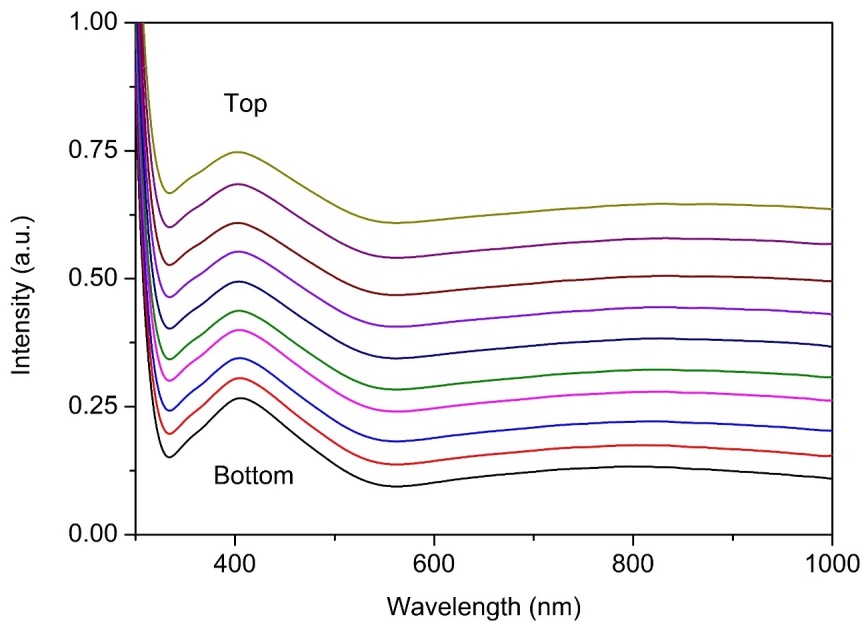


**Figure S30.** The position-dependent UV spectra of Ag NPs at different positions of solution with excess of NaCl (500 µL). 500 µL of NaCl was added into the Ag NPs colloid and diffused to the whole solution. The UV spectra were collected at different positions.


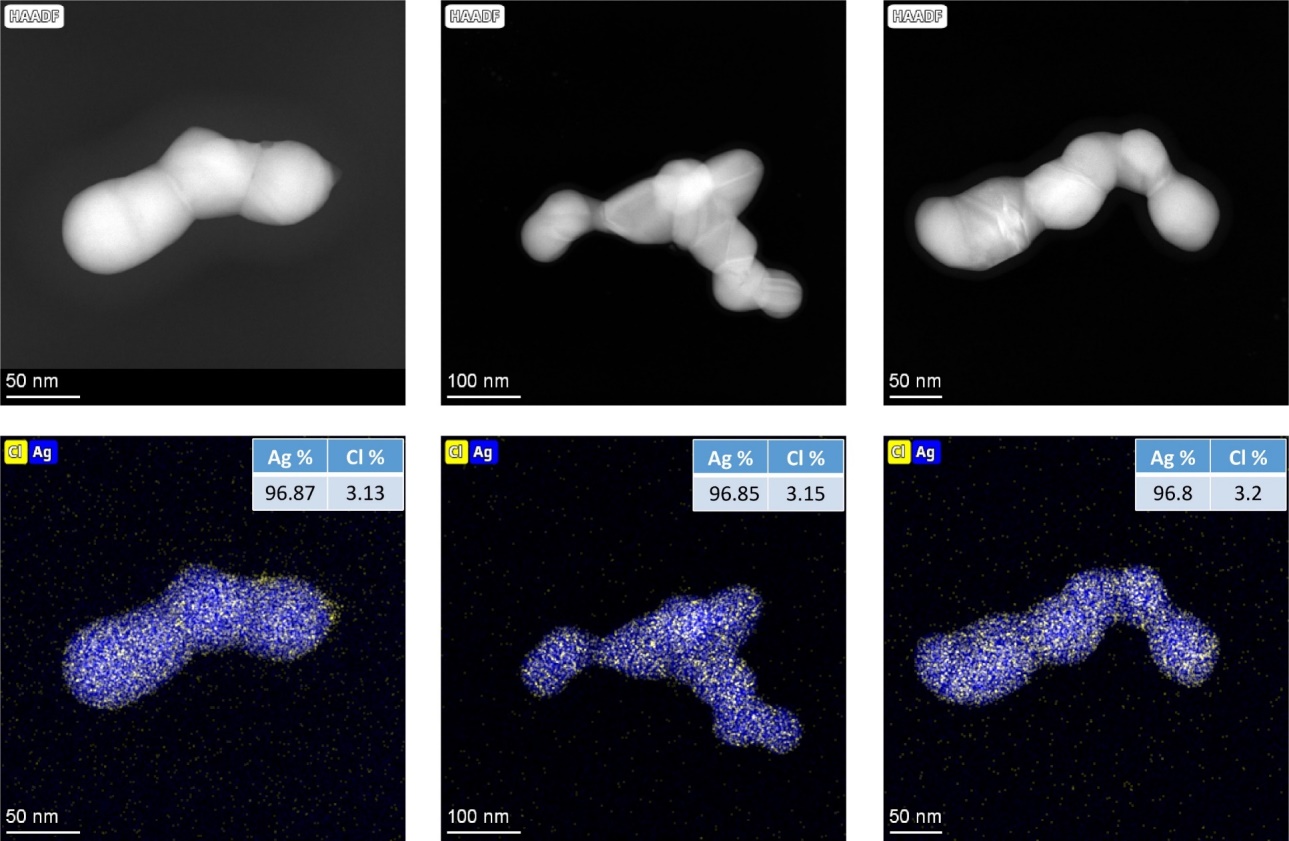


A

C

E

B

D

F

**Figure S31.** The EDS element mapping of Ag NPs at different positions of solution with excess of NaCl (500 µL). A-B) The Ag NPs at the top of the solution. C-D) The Ag NPs at the middle of the solution. E-F) The Ag NPs at the bottom of the solution. 500 µL of NaCl was added into the Ag NPs colloid. Ag NPs at the different positions were taken out and washed a couple of times to remove excess NaCl, then EDS characterization was performed.


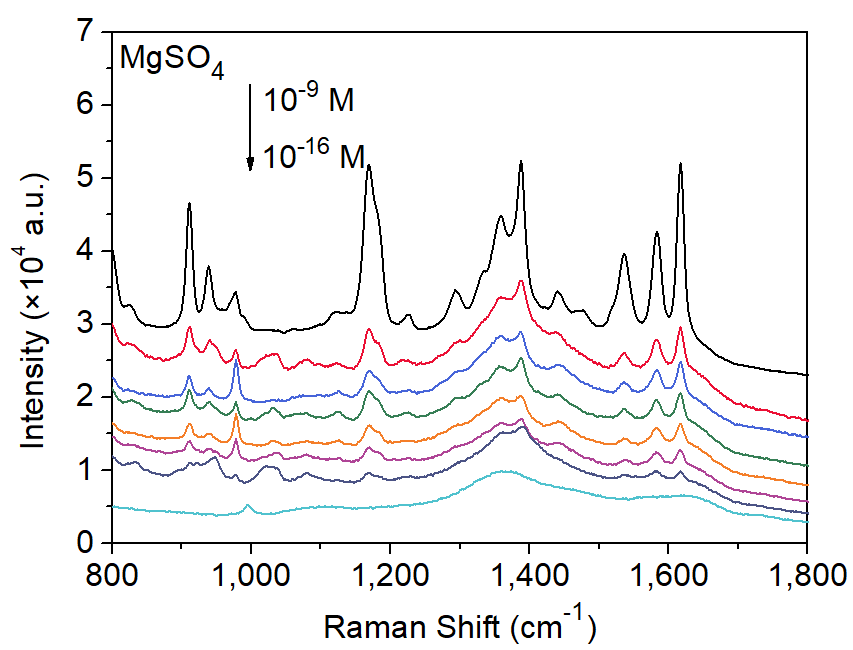


**Figure S32.** The SERS spectra of CV molecule in Ag colloid with MgSO_4_ as aggregation agent.

**REFERENCES**

(1) Wan, Y.; Guo, Z.; Jiang, X.; Fang, K.; Lu, X.; Zhang, Y.; Gu, N. Quasi-spherical Silver Nanoparticles: Aqueous Synthesis and Size Control by the Seed-mediated Lee-Meisel Method. *J. Colloid Interf. Sci.* **2013**, 394, 263-268.

(2) Zhang, L.; Li, X.; Liu, W.; Hao, R.; Jia, H.; Dai, Y.; Amin, M. U.; You, H.; Li, T.; Fang, J. Highly Active Au NP Microarray Films for Direct SERS Detection. *J. Mater. Chem. C* **2019**, 7, 15259-15268.

(3) You, H. J.; Fang, J. X.; Chen, F.; Zhu, C.; Song, X. P.; Ding, B. J. Concentration Profile within Diffusion Layer under Non-forced Hydrodynamic Conditions Measured by Michelson Interferometer. *Chem. Phys. Lett.* **2008**, 465, 131-135.

(4) Kneipp, K.; Yang, W.; Kneipp, H.; Perelman, L. T.; Feld, M. S. Single Molecule Detection Using Surface-Enhanced Raman Scattering. *Phys. Rev. Lett.* **1997**, 78, 1667-1670.

(5) Fleischmann, M.; Hendra, P. J.; Mcquillan. A. J. Raman Spectra of Pyridine Adsorbed at A Silver Electrode. *Chem. Phys. Lett.* **1974**, 257, 163-166.
